# Genetic redundancy fuels polygenic adaptation in *Drosophila*

**DOI:** 10.1371/journal.pbio.3000128

**Published:** 2019-02-04

**Authors:** Neda Barghi, Raymond Tobler, Viola Nolte, Ana Marija Jakšić, François Mallard, Kathrin Anna Otte, Marlies Dolezal, Thomas Taus, Robert Kofler, Christian Schlötterer

**Affiliations:** 1 Institut für Populationsgenetik, Vetmeduni Vienna, Vienna, Austria; 2 Vienna Graduate School of Population Genetics, Vetmeduni Vienna, Vienna, Austria; 3 Plattform Bioinformatik und Biostatistik, Institut für Populationsgenetik, Vetmeduni Vienna, Vienna, Austria; Georgia Institute of Technology, UNITED STATES

## Abstract

The genetic architecture of adaptive traits is of key importance to predict evolutionary responses. Most adaptive traits are polygenic—i.e., result from selection on a large number of genetic loci—but most molecularly characterized traits have a simple genetic basis. This discrepancy is best explained by the difficulty in detecting small allele frequency changes (AFCs) across many contributing loci. To resolve this, we use laboratory natural selection to detect signatures for selective sweeps and polygenic adaptation. We exposed 10 replicates of a *Drosophila simulans* population to a new temperature regime and uncovered a polygenic architecture of an adaptive trait with high genetic redundancy among beneficial alleles. We observed convergent responses for several phenotypes—e.g., fitness, metabolic rate, and fat content—and a strong polygenic response (99 selected alleles; mean *s* = 0.059). However, each of these selected alleles increased in frequency only in a subset of the evolving replicates. We discerned different evolutionary paradigms based on the heterogeneous genomic patterns among replicates. Redundancy and quantitative trait (QT) paradigms fitted the experimental data better than simulations assuming independent selective sweeps. Our results show that natural *D*. *simulans* populations harbor a vast reservoir of adaptive variation facilitating rapid evolutionary responses using multiple alternative genetic pathways converging at a new phenotypic optimum. This key property of beneficial alleles requires the modification of testing strategies in natural populations beyond the search for convergence on the molecular level.

## Abstract 2

Replicate populations of the fruit fly Drosophila simulans exposed to a novel temperature environment showed convergent evolution on the phenotypic level. On the genetic level, however, no convergence was observed. These results support the “trait optimum” paradigm as the predominant mode of adaptation.

## Author summary

It is widely assumed that adaptation is mainly polygenic, with the underlying frequency changes being so small that they are difficult to detect and characterize. Taking advantage of laboratory-based natural selection of replicated populations of the fruit fly *Drosophila simulans* exposed to a high temperature regime, we identified a polygenic response with many (99) selected alleles having pronounced allele frequency changes (AFCs). Despite phenotypic convergence across the 10 replicates, the genomic response was highly heterogeneous, with different subsets of the selected alleles contributing to the convergent phenotype in each of the replicate populations. The observed genetic redundancy not only indicates lack of genetic constraint but also demonstrates that multiple genetic pathways lead to convergent phenotypic outcomes.

## Introduction

Despite a long-standing interest, it is surprising how limited the understanding of the genetic architecture of adaptation is. The best characterized adaptive traits have a simple genetic basis such as pigmentation [1–3], lactose persistence [4], and resistance to viruses [5], insecticides [6], and malaria [7]. Population genetic tests for the identification of selected loci build on genomic signatures predicted for selective sweeps where selection targets independently spread in the population until they ultimately become fixed [8,9]. However, such simple traits are the exception rather than the rule, and most traits are polygenic, with many contributing loci [10,11]. Genome-wide association study (GWAS) analyses for traits such as human height [12,13], blood lipid levels [14], and basal metabolic rate [15]—which have identified many small-effect loci—provide strong evidence for the importance of the polygenic model. The allele frequency dynamics predicted for adaptive polygenic traits with a sufficiently large mutational target differ from those of selective sweeps [16,17]: subtle frequency changes at many small-effect loci, rather than large changes at few loci. The identification of the loci contributing to adaptive polygenic traits is, however, impeded by several challenges: (1) many causative variants identified in GWAS are deleterious, typically segregating at low frequencies [18]; (2) the adaptive role of many traits studied by quantitative trait locus (QTL) mapping and GWAS has not yet been convincingly demonstrated; (3) without replication and time series data, the analysis of extant populations may not be sufficiently powerful to distinguish between selective sweeps and polygenic adaptation. These challenges are probably best illustrated by human height, with the observed clinal variation in human populations being explained by selection on many loci with small effects [19,20]. Two recent analyses suggest, however, that the previously noted selection signal is better explained by population stratification, which was not sufficiently accounted for [21,22].

Due to these limitations, genomic selection signatures of polygenic adaptation to a new trait optimum have not received as much attention as selective sweep signatures. Once reliable alternative approaches are available to identify loci contributing to polygenic adaptive traits, it will be possible to address their importance for adaptive processes. Here, we demonstrate that replicated populations evolving in the same environment provide a novel approach to characterize adaptive loci. Specifically, we use whole-genome sequencing data from multiple time points in 10 replicated *Drosophila simulans* populations to identify selected alleles. We distinguish between the allele frequency dynamics of selective sweeps and polygenic adaptation by testing the redundancy of selected alleles during evolution in a new thermal environment. Genetic redundancy [23–25] is a key feature of polygenic traits with excess of beneficial variants; as a result, nonparallel genomic changes are expected in populations evolving to the same fitness optimum. We demonstrate that thermal adaptation in this species is highly polygenic and displays an unprecedented level of genetic redundancy, which has been predicted [25] but has rarely been conclusively demonstrated at the molecular level [25,26].

## Results

### Increased fitness in the evolved replicates

Temperature is a key environmental factor for all ectotherms, and the associated adaptive response in *Drosophila* involves many contributing loci [27]. To understand the genomic architecture of this canonical polygenic trait, we exposed 10 replicates (each with 1,000 individuals) from 202 *D*. *simulans* isofemale lines to a new hot temperature regime that cycled every 12 hours between 18 and 28°C, mimicking night and day. After more than 100 generations, we assessed the adaptive response of the 10 evolving replicates. We contrasted fecundity of the ancestral population with each of the evolved replicates after rearing all of them in the hot temperature regime. In agreement with previous results in *D*. *melanogaster* [28] and *simulans* [29], the evolved replicates had significantly higher fecundity, and therefore fitness, than the ancestral population (ANCOVA, *p* < 0.0001, S1A Fig).

### Reconstruction of the selected haplotype blocks

**Fig 1:**
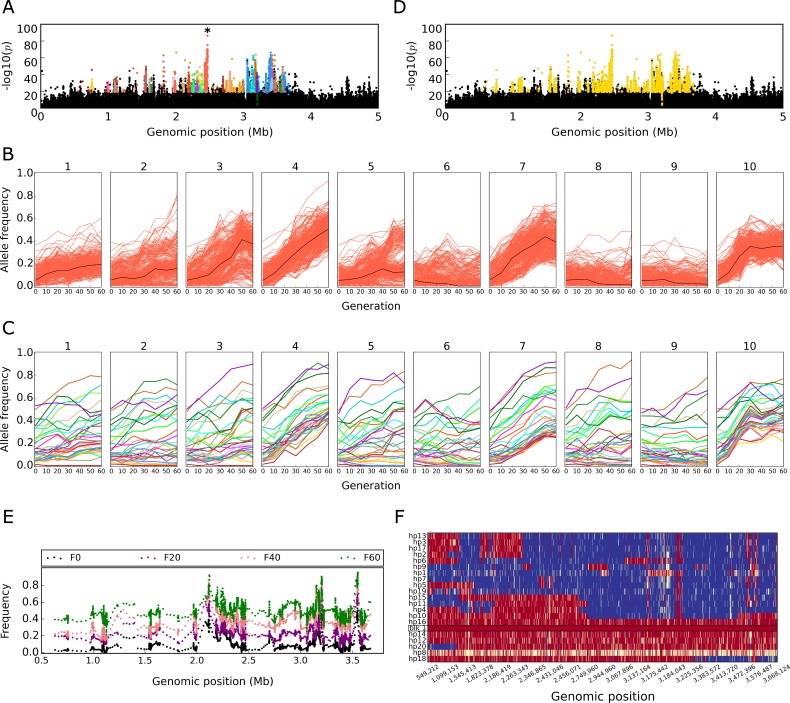
Reconstruction of selected haplotype block from Pool-Seq data. Manhattan plots (panel A, D) show negative log_10_ transformed *p*-values from CMH test contrasting the ancestral (F0) with evolved (F60) populations (Chromosome 2L: 1–5 Mb). SNPs with highly correlated allele frequencies across replicates and time points are clustered together with stringent clustering (panel A), and each cluster is indicated by a different color. SNPs in each cluster (e.g., the orange cluster marked with an asterisk in panel A) have correlated frequency trajectories across 60 generations in 10 replicates (panel B); the black line depicts the median allele frequency trajectory. (C) Trajectories of the median allele frequency of the correlated SNPs are shown for each of the clusters in this region (color code corresponds to panel A). Despite different starting frequencies, the median trajectories greatly resemble each other, suggesting that they are correlated and reflect a large selected genomic region. (D) Combining the adjacent short clusters with less stringent correlation identifies a haplotype block with weakly correlated SNPs. All SNPs, which cluster together, are used as markers for this selected haplotype block. (E) Time-resolved allele frequencies of marker SNPs for the haplotype block (panel D) are plotted along their genomic positions. Each dot indicates the mean frequency of five SNPs in overlapping windows (offset = 1 SNP). The time-resolved allele frequencies show a consistent increase across the entire haplotype block throughout the experiment. The data are from replicate 4. (F) The reconstructed haplotype block (panel D, E) is experimentally validated. Rows represent 20 phased haplotypes from evolved replicate 4. The reconstructed haplotype block is indicated by a black frame. Each column corresponds to a marker SNP, with red color indicating the character state of the haplotype block, blue the alternative allele, and yellow an unknown nucleotide. Data deposited in the Dryad Repository: https://doi.org/10.5061/dryad.rr137kn. CMH, Cochran-Mantel-Haenszel; SNP, single nucleotide polymorphism.

To characterize the genomic signature of adaptation in the evolved replicates, we generated replicated time series data by sequencing pools of individuals (Pool-Seq [30]) from the evolving replicates every 10th generation. After stringent filtering steps (Materials and methods), we obtained 5,090,460 single nucleotide polymorphisms (SNPs) on the major chromosomes (X, 2, and 3). We screened for SNPs with more pronounced allele frequency changes (AFCs) following 60 generations of evolution than were expected by genetic drift alone. After testing each replicate separately (Fisher’s exact test) and all replicates jointly (Cochran-Mantel-Haenszel [CMH] test) we obtained 52,199 candidate SNPs. The number of reported candidate SNPs is likely heavily inflated because these statistical tests assume independence of all SNPs, which is violated in our experimental population due to linkage disequilibrium between candidate SNPs [31,32] (in particular for candidate SNPs with a low starting frequency [33]). Reasoning that SNPs that are specific to selected haplotypes will have correlated allele frequencies across replicates and time points [33], we clustered SNPs by allele frequencies and reconstructed selected haplotype blocks (Fig 1; see Materials and methods “Reconstruction of the selected haplotype blocks (selected alleles)”). In total, we identified 99 haplotype blocks containing 23,835 SNPs, sized between 1.65 kb and 5 Mb (S2 Fig). To confirm the accuracy of the reconstructed haplotype blocks, we sequenced 100 haplotypes from five different evolved replicates and 189 ancestral haplotypes. We compared the marker SNPs of the identified haplotype blocks with the phased haplotypes to validate the inferred linkage patterns. Of the identified blocks, 96% (95 out of 99) were confirmed, demonstrating the robustness of our approach (see Fig 1F for an example, S1 Table). For the subsequent analyses, each haplotype block is considered a selected allele (see Materials and methods “Selected haplotype blocks: Selected alleles”).

### Phenotypic convergence of the evolved replicates

To determine the selected phenotypes driving the genomic selection signatures, we performed gene ontology (GO) enrichment analyses of genes overlapping with the SNP markers of the selected alleles. Consistent with there being many selected genes contributing to similar phenotypes, we detected significant enrichment in several GO categories, including oxidative phosphorylation, mitochondrial respiratory chain, ATP synthesis coupled electron transport, melanin biosynthesis process, monosaccharide transportation activity, DNA repair, and endopeptidase activity (S2 Table). Moreover, genes in the KEGG pathway oxidative phosphorylation were also significantly enriched (S3 Table).

**Fig 2:**
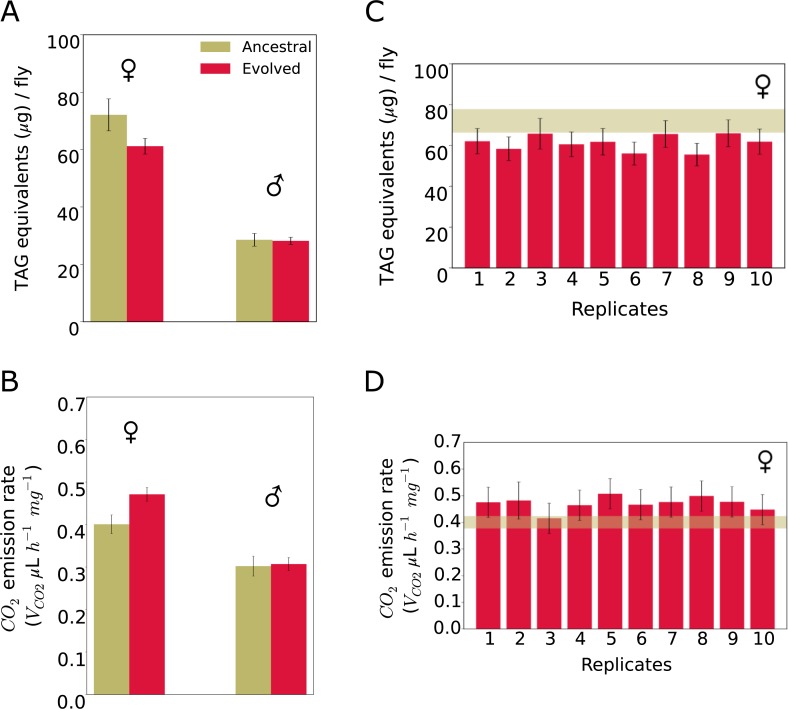
Adaptive response and phenotypic convergence in evolved replicates. (A) The amount of triglyceride as main constituent of body fat. Females of evolved replicates have significantly lower fat content than the ancestral population. Two-way ANOVA with interaction, Tukey’s HSD test *p =* 0.0007, (B) Resting metabolism measured by CO_2_ production as measure of resting metabolic rate. Females of evolved replicates produced significantly more CO_2_ than the ancestral population, two-way ANOVA with interaction, Tukey’s HSD test *p* < 0.0001. No significant difference was observed among males of the evolved replicates and the ancestral population (body fat: *p* = 0.7680; metabolic rate: *p* = 0.7405). Convergent evolution for fat content (panel C) and metabolic rate (panel D) for females of 10 evolved replicates. No significant difference was detected between the replicates (two-way ANOVA, Tukey’s HSD test *p* > 0.05). The bars show the least-squares means of the linear model, and error bars depict 95% confidence levels of least-squares means. The dark khaki horizontal bars show the 95% confidence levels of least-squares means in the ancestral population. Fat content and metabolic rate in males is shown in S1C and S1D Fig. Data deposited in the Dryad Repository: https://doi.org/10.5061/dryad.rr137kn. HSD, honest significant difference; TAG, triglyceride.

The GO enrichment analysis suggests that several phenotypes changed in response to the hot environment. We chose resting metabolic rate and fat content as high-level phenotypes for experimental validation because (1) they both reflect the enrichment for metabolic pathways (oxidative phosphorylation pathway), (2) fat content has been shown to respond to temperature [34], and (3) gene expression differences between hot- and cold-adapted *Drosophila* populations from Africa and Europe also reveal metabolic differences [29,35]. Consistent with the genomic signature, both fat content and metabolic rate differed between the ancestral population and the evolved replicates. Females in evolved replicates contained significantly less body fat than the ancestral population (*p* = 0.0007, Fig 2A) and had higher metabolic rates (*p* < 0.0001, Fig 2B), but no difference was noted for males (*p* > 0.7 for both traits, Fig 2A and 2B). No significant difference was detected for either of these two high-level phenotypes among the 10 evolved replicates (*p* > 0.05, Fig 2C and 2D). Thus, the 10 evolved replicates not only converged for fitness (S1B Fig) but also for other high-level phenotypes, i.e., fat content and resting metabolic rate.

### Genomic heterogeneity among the evolved replicates

Our experimental setting allowed us to empirically quantify several fundamental variables in adaptation genetics, including the starting frequency and distribution of selection coefficients (*s*) for beneficial alleles. Furthermore, the highly convergent phenotypic response across the replicates allowed us to investigate the fundamental question of whether adaptation to a specific stress is driven by the same alleles in all replicates or whether multiple alternative genetic routes are possible.

**Fig 3:**
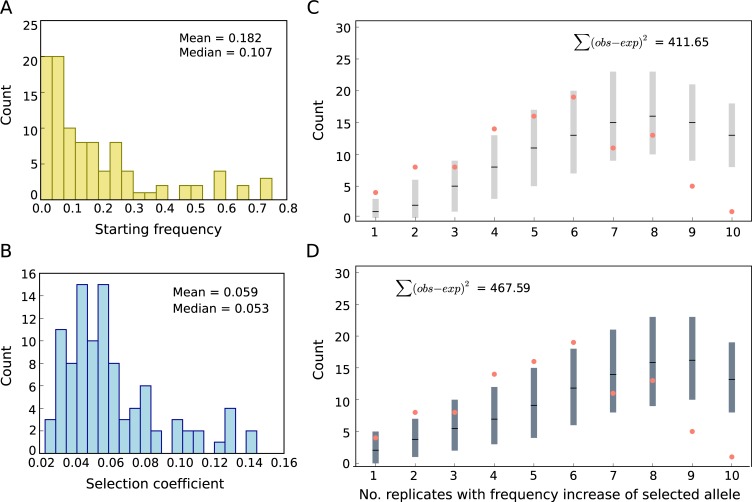
Characteristics of selected alleles: No match to the selective sweep paradigm. Distribution of (A) starting frequencies and (B) selection coefficients (*s*) of the selected alleles. The starting frequency of each selected allele has been estimated from the median frequency of all its marker SNPs. (C, D) The RFS from experimental data is indicated by salmon dots. The expected distribution of sweep paradigm with constant *s* across replicates without (C) and with (D) linkage using empirical *N*_*e*_ and locus-specific *s* (panel B) and starting frequency (panel A) was obtained from computer simulations. Light and dark grey bars show the 95% confidence interval of 1,000 iterations. Means are depicted in black lines in each bar. The difference between empirical (observed) and the mean of simulated (expected) data is shown as Σ(obs − exp)^2^. Data deposited in the Dryad Repository: https://doi.org/10.5061/dryad.rr137kn. RFS, replicate frequency spectrum; SNP, single nucleotide polymorphism.

**Fig 4:**
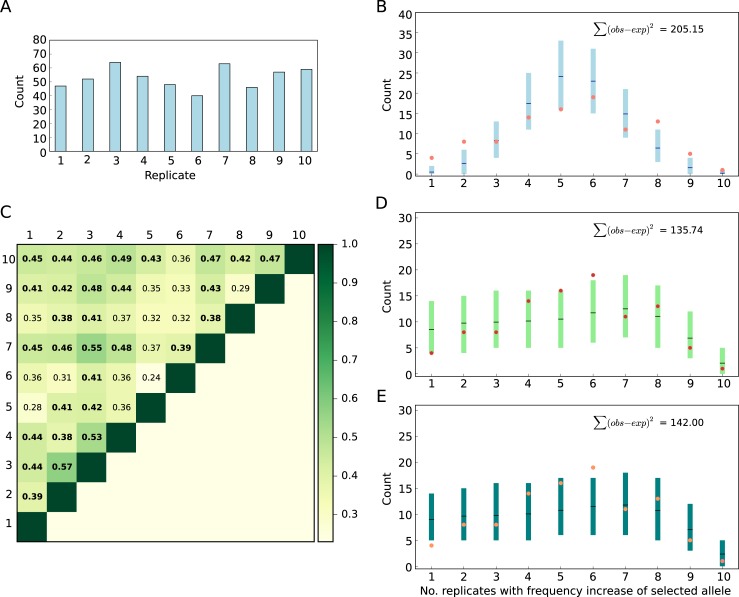
Adaptive genetic redundancy of selected alleles. (A) The number of selected alleles with ≥0.1 AFC (blue bars, average 53 loci/replicate) after 60 generations in each replicate. (B) Comparison of the empirical RFS (salmon) to the expected one under the assumption of genetic redundancy among alleles. 95% CI (blue) and means (black) were obtained by 1,000 iterations of delete-*d* jackknifing. (C) Pairwise Jaccard similarity indices among 10 evolved replicates. Indices significantly higher than expected by chance under the redundancy paradigm (panel B, Fig 5B) are shown in bold. Comparison of the empirical RFS (salmon) to the expected one assuming a QT paradigm without (panel D) and with (panel E) linkage after a change in trait optimum. 95% CI (light and dark green) were obtained by 1,000 iterations of forward simulations. The difference between empirical (observed) and the mean of simulated (expected) data, Σ(obs − exp)^2^, indicates a much better fit of the genetic redundancy and QT paradigm to the empirical data compared to sweep simulations (Fig 3C and 3D). Data deposited in the Dryad Repository: https://doi.org/10.5061/dryad.rr137kn. AFC, allele frequency change; RFS, replicate frequency spectrum.

Despite the high confidence in the reconstruction of selected haplotype blocks, i.e., selected alleles, the selected sequence variant(s) remain(s) unknown. Hence, we estimated the frequency of each selected allele using the median frequency of all its marker SNPs. Most of the selected alleles started from a low frequency in the ancestral population, but several alleles were rather common with frequencies up to 0.75 (Fig 3A). The strong selection coefficients of the selected alleles inferred across the replicates (min = 0.0229, max = 0.137 Fig 3B) suggest that a highly parallel genomic architecture could be expected [36–38]. However, we observed a highly heterogeneous response across the 10 replicates. A characteristic example is shown in Fig 1B and 1C, in which a selected allele undergoes a striking frequency change in some replicates but shows no change in others. We quantify the genomic heterogeneity among replicates using a new summary statistic, the replicate frequency spectrum (RFS), which reports the frequency distribution of replicates in which selected alleles increase in frequency. Indeed, using a ≥0.1 AFC cutoff, we find that most of the 99 selected alleles increase in only four to six replicates (Fig 3C, S3 Fig). Few alleles showed a selection response in only one or two replicates, and only a single allele increased in all 10 replicates (S3 Fig). On average, 53 selected alleles were identified per replicate (Fig 4A). To account for the influence of the starting allele frequencies in the ancestral population, we also used allele-specific frequency increase thresholds to determine selected alleles in evolved replicates (Materials and methods “Different approaches to determine the presence of selected alleles and their frequencies"), and the same trends were observed (S3 Fig), i.e., the frequency distribution of selected alleles is heterogenous across the replicates.

### Genomic heterogeneity does not match the sweep paradigm

We used the heterogeneous genomic pattern among replicates (S3 Fig) to discern several different adaptive scenarios (S4 Fig). Because the P-element is spreading in the ancestral population in our experiment [39,40], the observed heterogeneity among replicates might have been driven by the new replicate-specific P-element insertions. Nevertheless, a careful examination of P-element insertions showed that the observed insertions occurred at frequencies that were too low to explain the adaptive response (S4 Table).

**Fig 5:**
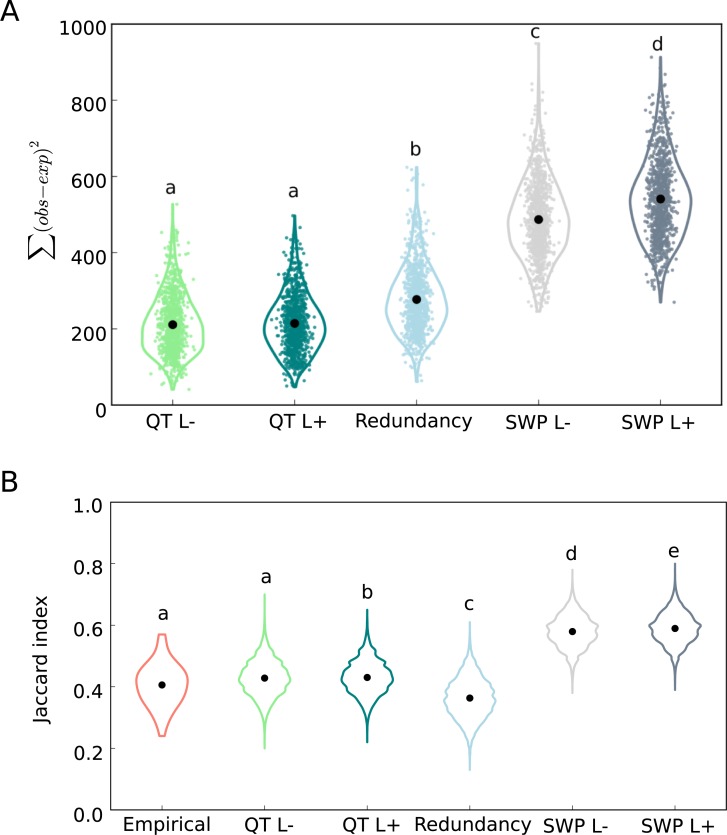
QT and redundancy paradigms fit the RFS of the empirical data better than selective sweep paradigm. (A) The difference between RFS of evolved replicates (observed) and that of the simulated (expected) data. For 1,000 iterations of each simulation, the difference between empirical and simulated RFS, Σ(obs − exp)^2^ is shown. The difference in RFS between evolved replicates and sweep simulations (SWP, without linkage: L− and with linkage: L+) are significantly higher than that of redundancy and QT paradigms (QT without linkage: L−; and with linkage: L+), One-way ANOVA, Tukey’s HSD test, *p* < 10^−5^. (B) Pairwise Jaccard indices in the empirical and simulated data. Replicates in the SWP L− and L+ simulations are significantly (*p* < 10^−5^) more similar than evolved replicates, while evolved replicates are significantly more similar than expected by chance, i.e., under the redundancy paradigm (*p* < 10^−5^). The Jaccard indices of replicates in QT simulations with (QT L+, *p* = 0.077) and without linkage (QT L−, *p* = 0.038) are more similar to the empirical data. Data deposited in the Dryad Repository: https://doi.org/10.5061/dryad.rr137kn. HSD, honest significant difference; QT, quantitative trait; RFS, replicate frequency spectrum; SWP, sweep.

With most selected alleles starting from a low frequency in the ancestral population (Fig 3A) and the moderate effective population size (*N*_e_) of the experimental populations (<300, S5 Table), the combined effects of selection and genetic drift could have contributed to the observed heterogeneity through differential loss of rare selected alleles across replicates. To test whether the observed heterogeneity among replicates could be explained by the interplay between selection and drift, we simulated evolution under a sweep paradigm with constant *s* (different among alleles but similar across replicates and time) but without linkage and epistasis. Using replicate-specific *N*_e_ estimates (S5 Table), and allele-specific starting frequencies and *s* (Fig 3A and 3B), we simulated 1,000 iterations of 99 independent alleles in 10 replicates across 60 generations. We detected some heterogeneity among replicates, but unlike the experimental data, most alleles spread in 7–9 replicates (Fig 3C). The difference between the RFS of simulated and experimental data was large (Fig 5A), and the similarities among replicates measured by the Jaccard index are significantly higher in simulated than experimental data (Fig 5B). The poor fit to the sweep paradigm (Fig 5A) could not be explained by how *s* was estimated or which threshold was used to detect a selected allele in a given replicate, as the same trends are seen regardless of which of the six estimation procedures (Materials and methods “Different approaches to determine the presence of selected alleles and their frequencies”) was used (S5 and S6 Figs).

To rule out the possibility that Hill-Robertson interference caused the observed heterogeneity among replicates, we included linkage in sweep simulations using information from 189 phased ancestral haplotypes. We simulated 99 selected alleles in 10 replicates of a population of 300 diploids (estimated *N*_*e*_) in 1,000 iterations with allele-specific starting frequencies and *s* (Fig 3A and 3B) and the recombination rate estimated from the ancestral haplotypes. The differences between the simulated and experimental data are still substantial (Figs 3D and 5A), and the simulated replicates are significantly more similar than the experimental replicates (Fig 5B) regardless of the threshold used to estimate *s* and detect selected alleles (S7 and S8 Figs). We conclude that the standard population genetic paradigm of selective sweeps, including Hill-Robertson interference, cannot explain the heterogeneous distribution of selected alleles among the replicated evolved populations.

### Adaptive genetic redundancy of the selected alleles

Other factors, such as linkage between selected and deleterious alleles, or frequency-dependent selection, could contribute to the observed replicate heterogeneity. Nevertheless, the combination of the striking phenotypic convergence (Fig 2C and 2D) and different subsets of selected alleles across replicates (S3 Fig) strongly suggests that the ancestral population contained more beneficial alleles than were needed to achieve optimum fitness [23–25], i.e., genetic redundancy. The simplest form of genetic redundancy is when all beneficial alleles have equal effects but not all of them are required in a given replicate to reach the fitness optimum. Under this scenario, different combinations of alleles can reach the fitness optimum in different replicates and subsequently produce a heterogeneous genomic pattern among them. We scrutinized genetic redundancy through jackknifing and randomly sampled a subset of the 99 selected alleles that matched the observed number of selected alleles for each replicate (Fig 4A). This simple paradigm of genetic redundancy fits the observed pattern of heterogeneity among replicates better than simulations based on the sweep paradigm; the difference in RFS between the simulated and experimental data is less than sweep paradigm (Figs 4B and 5A). Furthermore, the redundancy paradigm fits the experimental data better than the sweep paradigm regardless of the threshold used to detect selected alleles (S9 Fig).

Nevertheless, the combination of selected alleles shared across the replicates was significantly more similar in the experimental data (median Jaccard index = 0.41) than for randomly combined alleles of the redundancy paradigm (median Jaccard index = 0.36, *p* < 10^−5^, Fig 5B). Despite a significant difference, the experimental Jaccard index is only slightly higher than for random combinations of alleles. The higher similarity of the experimentally evolved replicates could simply be a consequence of alleles with higher starting frequency increasing in frequency in more replicates (S10 Fig). Thus, unlike a previous report of convergent adaptation in two tree species [41], we find no evidence for strong genetic constraints that limit the possible combination of beneficial alleles in our experimental populations.

Because our redundancy test did not model the frequency trajectory of selected alleles, we also simulated a quantitative trait (QT) with stabilizing selection after a shift in trait optimum. We assumed equal effect size for all 99 alleles in 10 replicates with starting frequencies matching the experimental data (Fig 3A) and simulated frequency trajectories of selected unlinked alleles in a population of 300 diploids for 60 generations. These simple QT simulations nicely matched the observed heterogeneity pattern (Fig 4D). The RFS difference between the simulated and experimental data was smaller than for other adaptive scenarios (Fig 5A), and the similarity among replicates in simulated data was not statistically different from the empirical data (Fig 5B). A similar fit was obtained when modeling linkage for the beneficial alleles in the QT paradigm, using the same linkage parameterization as in the sweep simulations (Figs 4E, 5A and 5B). Regardless of which frequency threshold was used to identify selected alleles, the difference in RFS between the experimental data and the simulated data under a QT without (S11 Fig) and with (S12 Fig) linkage was still less than sweep paradigm simulations (S8 Fig). Furthermore, the similarity among replicates in the simulated data was not different from the empirical data (S8 Fig).

## Discussion

Quantitative genetic theory assumes that, in populations close to the trait optimum, strong-effect alleles do not segregate at intermediate frequencies [42,43]. Thus, after shifts of the trait optimum, the phenotypic response is typically mediated by many small-effect alleles with no discernable change in allele frequency [16]. In our data, strongly selected alleles (mean *s* = 0.059) do occur at intermediate frequencies (Fig 3A, max = 0.75; mean = 0.182). This discrepancy may have several explanations, ranging from an ancestral population that has not reached the trait optimum to major impact of genetic drift and population structure and migration-selection balance in the ancestral population. The abundance of large-effect alleles contributing to adaptation also contradicts another theoretical prediction that polygenic trait adaptation is driven by alleles of small effect [44]. We addressed the possibility that the strong selection response in our experiment is driven by the combined effect of a large number of small-effect variants located on the selected allele: if a large number of randomly distributed variants contributes to the selected trait (i.e., infinitesimal model), the size of a selected genomic region should correlate with the number of contributing variants resulting in stronger selection for larger alleles. We tested this by regressing the estimated *s* of a given allele with its starting frequency, the number of replicates in which an allele increases in frequency (replicate frequency), and locus size (either physical or genetic distance). Because only the starting frequency (*p* < 1.11 × 10^−12^) and replicate frequency (*p* < 2.2 × 10^−16^) were significant, our data do not support a model with many randomly distributed targets of selection. These conclusions are robust to the choice of frequency increase threshold for determining replicate frequency and estimating *s* (S6 Table). Nevertheless, the results may differ with nonrandom distribution of selection targets.

**Fig 6:**
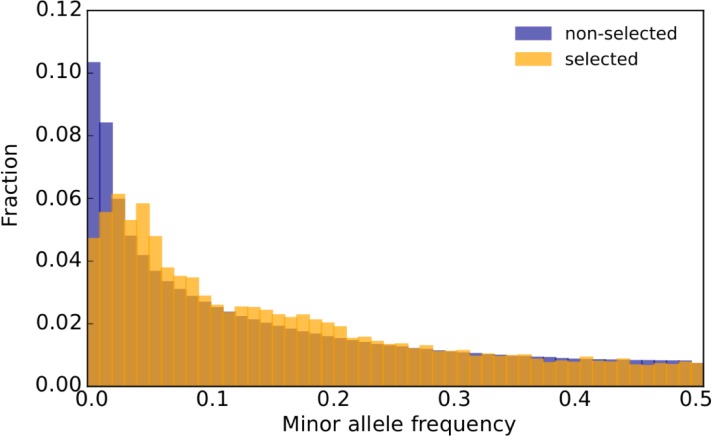
Selected alleles occur at higher frequency than neutral SNPs. The folded SFS of marker SNPs from selected alleles (23,835 SNPs) and nonselected SNPs (5,066,625 SNPs) in ancestral population is plotted. SNPs that were not identified as markers of selected alleles are considered nonselected SNPs. The distributions of these two SNP classes differ significantly (two-sided Kolmogorov-Smirnov test, ks value = 0.086, *p* < 10^−16^). Data deposited in the Dryad Repository: https://doi.org/10.5061/dryad.rr137kn. SFS, site frequency spectrum; SNP, single nucleotide polymorphism.

With 99 selected alleles and 202 founder isofemale lines, on average, every second founder carries a different selected allele. This implies that natural *D*. *simulans* populations harbor vast reservoirs of variants capable of contributing to temperature adaptation and different combinations of these variants result in similar phenotypic changes. Thus, beneficial alleles tend to segregate at higher frequencies than neutral SNPs in our experimental population (Fig 6), suggesting a role for balancing selection—possibly driven by seasonal temperature changes [45].

Is the adaptive genetic redundancy of temperature adaptation an exception, or do more traits have a similar genetic basis? The empirical evidence for adaptive genetic redundancy is extremely sparse, but this probably reflects a bias toward methodologies that search for convergent genetic changes. Studies of genomics of adaptation in yeast [46] and *Drosophila* [36,37] have identified repeatable genomic signatures in replicate populations. However, these populations have either started from fewer lines, whereby beneficial alleles had higher starting frequencies [46] or had been maintained in lab for a very long time [36,37]. Indeed, recent studies that used freshly collected individuals have identified more heterogeneous genomic responses among replicate populations [26]. Examples for adaptive genetic redundancy include de novo mutations in experimental *Escherichia coli* populations [47], pigmentation in African *Drosophila* in which different genes contribute to the same dark phenotype [48], desiccation resistance [26], and the hemoglobin oxygen affinity mediated by different amino acid substitutions in 56 avian taxa [49]. Truncating selection studies in *Drosophila* [50] and corn [51] reporting rapid phenotypic responses despite a small number of founders also indirectly support the presence of abundant genetic redundancy in natural populations. The abundance of large-effect beneficial alleles segregating in natural populations suggests that more alleles are segregating than needed to reach the fitness optimum, and ultimately many alternative genetic pathways can be used. Thus, the genomic signature of adaptation could differ among natural populations exposed to a similar environment, and genome scans for convergent genomic signatures across populations are less likely to succeed for such QTs.

## Materials and methods

### *D*. *simulans* experimental population and the selection regime

Ten replicate populations were set up using 202 isofemale lines (inseminated females maintained by brother-sister mating) from a natural *D*. *simulans* population collected in Tallahassee, Florida [52]. Each isofemale line represents a separate population of 40–50 individuals maintained in the laboratory for about nine generations before the replicates for experimental evolution were set up. Five mated females from each isofemale line were used to establish each of 10 replicates. These replicates were maintained in a new hot environment in which both temperature and light cycled every 12 hours between 18°C and 28°C, corresponding to night and day. The replicates had a census population size of 1,000 and about a 50:50 sex ratio. The flies in each replicate were equally distributed across five 300 mL bottles containing 70 mL of standard *Drosophila* medium.

### Genome sequencing and mapping of sequence reads

Genomic DNA was extracted for all replicates at generation 0 (females only) and all evolved replicates in 10 generation intervals until generation 60 (mixed sexes). The replicates at generation 0 will be referred to as “ancestral population” hereafter. Details of DNA extraction and library preparation are provided in S7A Table. Sequencing of paired-end 100 bp reads resulted in an average genome-wide sequence coverage of approximately 216× for each ancestral and approximately 103× for each evolved replicate. Trimming, mapping, and filtering of reads was performed as described in [52].

### SNP calling

SNPs were called from replicates of the ancestral population; in brief, SNPs with base quality of 40 in at least one of the 10 ancestral replicates were selected for further analyses. To improve the reliability of the pipeline, the polymorphic sites in the upper 1% and lower 1% tails of the coverage distribution (i.e., ≥423× and ≤30×, respectively; upper tail based on the library with the highest sequencing depth; lower tail estimated from total coverage of all ancestral and evolved replicates at generation 60, S8 Table), and minor alleles with coverage less than 10 reads were removed. Furthermore, we masked repeats (transposable elements [TEs] were annotated using the pipeline described in [53]), and 5-bp regions flanking indels (identified by PoPoolation2 [54]: using function *identify-genomic-indel-regions*.*pl* with options—*indel-window 5—min-count 167*). The minimum read count cutoff corresponds to 2% of the average coverage across all ancestral and evolved replicates. We further masked 200-bp flanking the SNPs specific to autosomal genes translocated to the Y chromosome [55]. After these filtering steps, the remaining 5,090,460 SNPs on the major chromosomes (X, 2, and 3) were used for subsequent analyses. For these SNPs, we determined the allele frequencies using only reads with a base quality score of at least 20 at the SNP position.

### Inference of candidate SNPs

To identify SNPs with pronounced AFCs, Fisher’s exact and CMH tests were used. First, we contrasted the ancestral and the evolved replicates at generation 60 using the CMH test to identify SNPs with a consistent frequency change across replicates (using PoPoolation2, function *cmh-test*.*pl*). Second, the pronounced AFCs specific to each replicate were determined by contrasting each ancestral replicate with the corresponding evolved replicate at generation 60 (e.g., ancestral replicate 5 with evolved replicate 5) using Fisher’s exact test (PoPoolation2, function *fisher-test*.*pl*: option—*min-count 5*). For the parameters of *fisher-test*.*pl*, the minimum SNP coverage for each replicate was set to 5% of the average coverage of ancestral and evolved (generation 60) samples. For the maximum coverage, the upper 1% tail of the coverage distribution in the sample with the highest sequencing depth was chosen (S8 Table). In total, 10 Fisher exact tests were performed.

Neither the CMH nor the Fisher’s exact test accounts for drift. Thus, to determine the candidate SNPs whose AFCs were higher than expected under drift, neutral Wright-Fisher simulations were performed with Nest [56] (function *wf*.*traj*) assuming independence among SNPs. As a first step for the neutral simulations, *N*_e_ was estimated for each of the replicates. We used windows of 1,000 SNPs based on AFCs between the ancestral and evolved replicates at generation 60 for autosomes (each chromosome separately) and the X chromosome using Nest (function *estimateWndNe*, method *Np*.*planI*). We averaged the medians of the *N*_e_ estimates across replicates; the estimated *N*_e_ was 291 for autosomes and 262 for the X chromosome (S5 Table), and we used these estimates of *N*_e_ to perform simulations to compute false discovery rate–corrected q-values of the CMH test. We computed false discovery rate–corrected q-values for the Fisher’s exact test by performing neutral simulation using replicate-specific *N*_e_ estimates for autosomes (S5 Table) and a *N*_e_ of 262 for the X chromosome. The simulation parameters (i.e., number of SNPs, allele frequencies in the ancestral replicates, sequence coverage of replicates, and the number of replicates and generations) matched the experimental data. Candidate SNPs were inferred based on an empirical CMH/Fisher’s exact test cutoff (q ≤ 0.05) using neutral simulations. After correction, we obtained 47,532 candidate SNPs across replicates (CMH test), and 4,667 additional SNPs deviated from neutral expectations in at least one of the replicates (Fisher’s exact test). This number of candidate SNPs is still heavily inflated due to the considerable linkage expected in our experiment.

### Reconstruction of the selected haplotype blocks (selected alleles)

Because only few recombination events occur during 60 generations of evolve and resequence (E&R) in *Drosophila*, selected variants typically occur on rather large haplotype blocks [32,57]. While some methods have been proposed to reconstruct haplotypes from Pool-Seq data [46,58,59], they are restricted to cases in which most of the founder haplotypes are known. Although we have sequenced 189 phased haplotypes from the ancestral population, this number accounts for only about 25% of the founder chromosomes (the 202 isofemale lines used for establishing the ancestral population were not inbred). Therefore, we identified haplotype blocks carrying the beneficial mutation using a modification of a recently published approach [33]. We reasoned that SNPs specific to selected haplotypes have correlated allele frequencies across replicates and time points [33]. Thus, we clustered SNPs by allele frequencies and shifted our focus from candidate SNPs to selected haplotype blocks.

First, we grouped candidate SNPs together with stringent clustering (minimum average Pearson’s correlation coefficient of 0.75 among SNPs). Candidate SNPs (q ≤ 0.05) inferred from both CMH and Fisher’s exact tests were combined and used for the selected haplotype block reconstruction; the candidate SNPs from the CMH test were polarized based on the rising allele, and only SNPs with a frequency increase of ≥0.2 (between any time points from F0 to F60) in at least two replicates were retained. Additionally, all candidate SNPs from Fisher’s exact tests were polarized based on the rising allele and used for block reconstruction. The allele frequencies of these candidate SNPs were transformed (arcsine of the square root, using *numpy*.*arcsin* and *numpy*.*sqrt* functions in Python) and standardized, i.e., centered to mean and scaled to unit variance (using function *sklearn*.*preprocessing*.*scale* in Python). The left and right arms of Chromosomes 2 and 3 were concatenated for this analysis, because some haplotype blocks overlap the centromere. All pairwise Pearson’s correlation coefficients were computed among SNPs in sliding windows of 1 Mb with a step size of 500 kb. Each window should have at least a minimum of 20 candidate SNPs. The correlation matrix was converted to Euclidean distance, d = √2(1 − r), and entered in a distance matrix (using function *scipy*.*spatial*.*distance*.*squareform* in Python), which was used for hierarchical clustering (using function *scipy*.*cluster*.*hierarchy*.*linkage* in Python). SNPs with the minimum average Pearson’s correlation coefficient of 0.75 were classified into a cluster. Clusters obtained from overlapping windows were merged if they shared at least five SNPs. Only clusters with more than 20 SNPs were retained.

We identified many relatively short clusters (Fig 1A and 1B). Recombination, either in the ancestral population or during the experiment, results in less strongly correlated allele frequency trajectories. Hence, several adjacent clusters show very similar frequency change across replicates (Fig 1C). Therefore, all the SNPs in clusters with average correlation of 0.75 (see above) were used for another round of clustering (similar to above), but SNPs with the minimum average Pearson’s correlation coefficient of 0.35 were classified into a cluster. Similar to above, clusters that share at least five SNPs in overlapping windows were merged. The importance of combining clusters with correlated allele frequency trajectories into a haplotype block is evident from the presence of several distinct peaks in the genomic region underlying a haplotype block (Fig 1A). Without information about the correlation, a naïve interpretation may have been that several independent alleles were selected.

### Experimental inference of haplotypes

We experimentally determined the full chromosomal haplotypes by crossing males from the evolved replicates with virgin females from the reference strain M252 [60] (Genbank BioSample SAMN02713493). In total, we obtained 100 haplotypes from five evolved replicates (20 from replicate 1 at F88, 36 from replicate 3 at F103, 20 from replicate 4 at F88, 12 from replicate 7 at F103, and 12 from replicate 10 at F103). A single F1 female of each cross was used for DNA extraction and sequencing. Details of DNA extraction and library preparation are provided in S7B Table. Trimming and mapping the sequencing reads and filtering of mapped reads was performed as described in [52]. Haplotypes were called from F1 individuals as described in [61]. The F1 genotype was compared to the reference strain (Genbank assembly accession: GCA_000820565.1), and the parental alleles were determined. Haplotype base calling was performed for positions that had a coverage larger than 10 and less than the maximum 2% coverage of the respective library.

### Validation of reconstructed haplotype blocks using phased haplotypes

To demonstrate the robustness of the haplotype reconstruction approach, we compared the allelic states of SNPs in each reconstructed block to 100 phased haplotypes from five different evolved replicates and also 189 phased haplotypes from the ancestral population [62]. We accounted for the presence of ambiguous alleles (very low or high coverage) in phased haplotypes by allowing up to 20% missing or mismatched SNPs to consider a phased haplotype to contain a reconstructed block (i.e., ≥80% sequence identity). Of the reconstructed haplotype blocks, 84% and 82% were present in the evolved and ancestral haplotypes, respectively (see Fig 1F for an example, S1 Table). Blocks that could not be validated either have low frequency in the ancestral population or increased in frequency in a evolved replicate with no available phased haplotype.

### Selected haplotype blocks: Selected alleles

Typically, multiple haplotypes are associated with a putative selection target, in particular when it occurs at higher frequency in the ancestral population. Because we cannot identify the selected mutation, we use the term “selected allele” in the subsequent analyses to refer to selected haplotype blocks (Fig 1D and 1E), i.e., describing a suite of haplotype blocks carrying the target(s) of selection. Due to the limited mapping resolution in our experiment, we cannot distinguish whether the selection signature of a selected allele is generated by multiple selected targets (allelic heterogeneity) or a single target of selection. However, we addressed the possibility that the selection response in our experiment is driven by the combined effect of a large number of small-effect alleles located on the selected haplotype blocks (Materials and methods: “Identification of factors affecting *s*”).

### Different approaches to determine the presence of selected alleles and their frequencies

Despite selected haplotype blocks being reconstructed with high confidence, the selected sequence variant remains unknown. Because estimates of the selection coefficient (*s*) and the results of computer simulations under the different selection regimes critically depend on the allele frequency of the selection target, we used six different methods to estimate the frequency of the selected allele. In the ancestral and evolved populations, the frequency of selection targets is estimated by the median frequency of all SNPs characterizing a selected allele (please note that we redefine selected alleles for method 5 and 6). Furthermore, our analyses require the distinction between selection targets increasing in frequency (i.e., contributing to adaptation) and those not contributing to adaptation.

#### Methods 1 and 2

Method 1 and method 2 use an ad hoc cutoff for AFC in the contrast between the ancestral population and the evolved replicates at generation 60. Thus, replicates with at least a 0.1 (method 1) or 0.2 (method 2) frequency increase of selected alleles after 60 generations were considered to have alleles contributing to adaptation and consequently were included in the calculation of *s* (see below: “Inference of selection coefficient”).

#### Methods 3 and 4

Because the AFC for selected and neutral alleles depends strongly on the allele frequency in the ancestral population, methods 3 and 4 use an allele-specific frequency change threshold, which depends on the frequency in the ancestral population. We performed selective sweep simulations to determine the expected frequency increase under selection for each allele. For each selected allele, *s* was computed for all 10 replicates (“Inference of selection coefficient”), and the highest *s* was used for forward Wright-Fisher simulations. A total of 1,000 simulations were performed for each allele using PoolSeq [63] (function *wf*.*traj*) using replicate-specific *N*_*e*_ (S5 Table) and starting frequency. The frequency increase threshold for each selected allele was the lower 5% (method 3) and 10% (method 4) tail of the AFC distribution under forward Wright-Fisher sweep simulations.

#### Method 5

Because the selected allele includes a broad genomic region and the correlated SNPs have different starting frequencies (Fig 1, Materials and methods “Reconstruction of the selected haplotype blocks (selected alleles)”), the allele frequency estimates may be biased by a too broad definition of the selected allele. To avoid this bias, we assumed that the region with the highest *s* in a selected allele contains the target(s) of selection: each selected allele was divided into several regions (clustering with stringent correlation coefficient of 0.75), and for each region, the median *s* across the replicates with a frequency increase ≥0.1 was computed, similar to method 1. The region with the largest *s* was chosen as the “core region” of the selected allele, and *s* (S5K Fig) and the starting frequency (S5M Fig) of the core region were used as the representative of the selected allele.

#### Method 6

Similar to method 5, core regions of each selected allele were identified, and for each region, the median *s* (S5N Fig) and the starting frequency (S5P Fig) across the replicates with a frequency increase ≥0.2 were computed.

### Inference of selection coefficient

Selection coefficients (*s*) were estimated assuming codominance (*h* = 0.5) with PoolSeq [63] (using the function *estimateSH*), which uses time series allele frequency data to infer *s*. To account for the uncertainty about the selection targets, we used six different approaches to determine whether an allele contributed to adaptation in a replicate (see above, methods 1–6). For replicates with frequency increase ≥threshold after 60 generations (determined by methods 1–6), *s* was estimated, and the median *s* across the corresponding replicates is reported as *s* of the selected allele (Fig 3B: method 1; S5B Fig: method 2; S5E Fig: method 3; S5H Fig: method 4; S5K Fig: method 5; S5N Fig: method 6).

Furthermore, we checked whether reporting the median *s* of those replicates with frequency increase ≥threshold (methods 1–6) results in a biased *s* estimate. Thus, rather than reporting the median *s*, we also reported *s* separately for each replicate. We show the replicate-specific *s* for all approaches of estimating *s* (S5A Fig: method 1; S5C Fig: method 2; S5F Fig: method 3; S5I Fig: method 4; S5L Fig: method 5; S5O Fig: method 6). Overall, all methods agree very well (S5 Fig), but an AFC ≥0.1 (method 1) and ≥5% allele-specific frequency change (method 3) are the most conservative methods resulting in the lowest *s*.

### Identification of factors affecting *s*

We identified factors influencing *s* by fitting a linear model with three fixed continuous effects (starting frequency [i.e., median starting frequency of all SNPs characteristic to the selected allele: *p*_0_]; replicate frequency [i.e., the number of replicates in which a specific allele increases ≥threshold in frequency]; and locus size [i.e., the genomic region corresponding to an allele: *size*]) and interaction between replicate frequency and *p*_0_. We estimated *s* using methods 1, 3, and 4 (Materials and methods: “Inference of selection coefficient”). *s* and replicate frequency were log10-transformed (*log10* function in R), and the square root of *p*_0_ was arcsin-transformed (*asin* and *sqrt* functions in R). We accounted for nonlinearity of *p*_0_ by adding a quadratic term (squared *p*_0_) to the model. The interaction between replicate frequency and *p*_0_ was not significant and was therefore dropped from the model (*s*_ijklm_ ~ μ + *p*_0i_ + replicate frequency_j_ + *p*_0_^2^_k_ + size_l_ + error_ijklm_). The data met the assumptions of normality of the residuals and homogeneity of variance. Moreover, we used the *D*. *simulans* recombination map (Dsim_recombination_map_LOESS_100kb_1.txt in [62]) to convert locus size (*size*) into genetic distance: *genetic distance*. We then performed the regression model (*s*_ijklm_ ~ μ + *p*_0i_ + replicate frequency_j_ + *p*_0_^2^_k_ + genetic distance_l_ + error_ijklm_). We did not find a significant contribution of locus size or genetic distance (S6A Table). Similar results were obtained independent of the method for *s* estimation (S6B Table).

### Contrasting selective sweep and QT paradigms

We observed a highly heterogeneous response across the 10 evolved replicates; most of the 99 selected alleles increase in only four to six replicates (S3 Fig). We used this heterogeneous pattern among replicates to discern several different adaptive scenarios (S4 Fig).

To compare different evolutionary paradigms (sections A–E below correspond to scenarios A–E in S4 Fig) to the empirical data, we used the RFS—i.e., the frequency distribution of replicates in which selected alleles increase in frequency—as a summary statistic to measure the fit between the simulated and observed data. We determined the RFS using different thresholds of frequency increase (method 1–6) to identify replicates with a selected allele. These thresholds are specified in S4 Fig with different colors.

Because simulations of the QT paradigm (sections C–E below, C–E in S4 Fig) require additional parameters, which cannot be estimated from the data (e.g., distance to the trait optimum), we did not perform simulations for all methods to estimate the frequencies of selected alleles. We used only three different thresholds based on method 1, 3, and 4 to identify selected alleles in each replicate.

In each evolutionary scenario summarized in section A–E, 1,000 sets of 10 replicates (similar to empirical data) were simulated, RFS was determined for each set, and mean and 95% CI were computed for each category of RFS (i.e., 1 to 10 replicates). For each simulation, we compared RFS of the empirical data (observed) and the mean RFS of simulations (expected) using Σ(obs − exp)^2^. We compared Σ(obs − exp)^2^ of different simulations using one-way ANOVA with Tukey’s honest significant difference (HSD).

We also estimated the similarity among replicates by calculating the Jaccard index of all pairwise combinations in evolved replicates using the same three thresholds used for QT simulations (method 1, 3, 4; see above). Pairwise Jaccard indices were also computed for each simulation set of 10 replicates for all evolutionary scenarios in A–E using the same three frequency change thresholds: methods 1, 3, and 4 (Figs 5 and S8).

#### A. Sweep paradigm with a constant *s* across replicates and no linkage

Because drift in small populations can result in considerable heterogeneity among replicates, we used computer simulations to test whether the observed heterogeneity among replicates can be explained by the combined effect of the selective sweep and drift. A total of 1,000 sets of forward Wright-Fisher simulations were performed using PoolSeq [63](function *wf*.*traj*). In each set, 99 independent alleles (matching the observed number of selected alleles) were simulated for 60 generations in 10 replicates using the allele-specific starting frequency, replicate-specific *N*_*e*_ (S5 Table), and allele-specific *s* assuming no linkage and epistasis (A1–6 in S4 Fig). Six different methods were used to estimate the frequency of the selected allele and its associated selection coefficient (S5 Fig). Selected alleles on autosomes and the X chromosome were simulated separately using *N*_*e*_ estimated for autosomes and the X chromosome, respectively (S5 Table). The results for method 1 are shown in Fig 3C, and results of other methods are provided in Supporting Information (S6 Fig).

Twenty of the 99 selected alleles in the experimental evolution increased ≥0.1 in frequency in one to three replicates only. These alleles have low starting frequencies (S10 Fig) and the highest estimated *s* (S10 Fig). This may imply that we overestimated *s* for these alleles (winner’s curse). To exclude the possibility that an overestimate of *s* for alleles that increased in frequency only in a small number of replicates affects the outcome of simulations, we repeated the simulations (S6F Fig) with the default parameters in A2 (S4 Fig) using only those 79 alleles that increased ≥0.1 in frequency in more than four replicates (A7 in S4 Fig).

Independent of estimated *s*, the RFS difference between empirical and simulated data was higher than simulations of other evolutionary scenarios, and we could not obtain a good fit to the empirical RFS (S6 and S8 Figs).

#### B. Sweep paradigm with linkage and a constant *s* across replicates

We simulated a sweep paradigm assuming linkage among selected alleles to rule out that Hill-Robertson interference caused the genetic heterogeneity among the replicates. We used 189 individually sequenced haplotypes from the ancestral population [62] for the simulations. We simulated 99 selected alleles (the number of selected alleles in the experimental data) in 10 replicates of a population of 300 diploids (corresponding to the estimated *N*_*e*_) in 1,000 iterations for 60 generations with the recombination rate estimated from the haplotypes (Dsim_recombination_map_LOESS_100kb_1.txt in [62]). Similar to the simulations of selective sweep without linkage, we used six different methods (B1–6 in S4 Fig) to infer the frequencies of the selected allele and the associated selection coefficient (*s*). We also repeated the simulations (S7F Fig) with the default parameters in B2 (S4 Fig) using only those 79 alleles that increased ≥0.1 in frequency in more than four replicates (B7 in S4 Fig). The position and starting frequency of the selected target was chosen to match the position of one of the characteristic SNPs in the selected allele and the starting frequency of the selected allele. Simulations were performed using function *w* in MimicrEE2 (version mim2-v193), which uses haplotype information and also accounts for the differences between the X chromosome and autosomes [64]. Simulations were performed assuming a balanced sex ratio. The results for method 1 are shown in Fig 3D, and the results obtained from the other methods are given in Supporting Information (S7 Fig).

Similar to sweep simulations in section A, the RFS difference between empirical and simulated data was high, and we did not obtain a good fit to the empirical RFS (S7 and S8 Figs).

#### C. Genetic redundancy paradigm

One prediction of the QT paradigm with stabilizing selection is that the same trait optimum can be obtained by different combinations of contributing alleles (genetic redundancy). Before simulating the trajectories of the selected alleles, we first tested genetic redundancy.

Independent of the method used to estimate the frequency of the selected allele (and thus the method to determine the threshold for allele frequency increase after 60 generations), a different number of selected alleles was detected among the replicate populations (S3 Fig). Nevertheless, all replicates converged for the high-level phenotypes: fitness, fat content, and resting metabolic rate (Figs 2C, 2D and S1B). Therefore, we tested whether the frequency distribution of the selected alleles among replicates of our experiment fits a paradigm of full genetic redundancy. Assuming that all alleles are functionally equivalent, we generated 1,000 sets each consisting of 10 replicates using delete-*d* jackknifing. In each set, the number of selected alleles for each replicate matched our observations (using method 1, Fig 4A) but was randomly drawn (without replacement) from the total of 99 selected alleles (C in S4 Fig). Furthermore, we performed delete-*d* jackknifing using the number of selected alleles in each replicate computed using method 3 and 4 (S9 Fig).

#### D. QT paradigm without linkage

To determine whether the genomic heterogeneity among the replicates of the empirical data are compatible with the QT paradigm, we simulated frequency trajectories of alleles contributing to a QT after a change in trait optimum (D in S4 Fig). Using forward simulations in a population with 300 diploids, we simulated 1,000 iterations of a QT in 10 replicates for 60 generations with 99 unlinked contributing alleles having the same starting frequency as the selected alleles in the empirical data (Fig 3A). We assumed random mating, and all contributing alleles had equal effects. The phenotypic values of the QT were computed and mapped to fitness using a Gaussian fitness function in which the fitness ranged between 0.5 and 4.5, and the mean phenotype optimum was set to 0.6 with standard deviation of 0.3 (S13A Fig). Simulations were performed using the Python script of [65] (*frequencyAt-pheno-quantitative*.*py*).

#### E. QT paradigm with linkage

We simulated adaptation of a QT to a new trait optimum in a diploid population of 300 individuals assuming linkage among the selected alleles. Similar to the QT paradigm without linkage, the phenotypic values of the trait were mapped to fitness using a Gaussian fitness function. We performed 1,000 iterations of forward simulations of a QT in 10 replicates with 99 contributing alleles with equal effects (E in S4 Fig). The position and starting frequency of the selected target was chosen to match the position of one of the characteristic SNPs in the selected allele and the starting frequency of the selected allele in the ancestral population (Fig 3A). The optimum phenotype was set at −1.3 with a standard deviation of 1.2, and fitness ranged between 0.5 and 4.5 (S13B Fig). We recorded the population allele frequencies for 60 generations. The selected alleles were recombined according to the *D*. *simulans* recombination map (Dsim_recombination_map_LOESS_100kb_1.txt in [62]). Simulations were performed assuming a balanced sex ratio using function *qff* of MimicrEE2 (version mim2-v193) [64].

### Identification of TEs

Because the ancestral population experienced the invasion of a P-element [39,40], it may be possible that new P-element insertions were driving adaptation and thus contribute to the observed heterogeneity among replicates. Adaptation driven by the P-element should result in a frequency increase of the P-element, which matches the frequency increase of the corresponding allele in a given replicate. Hence, we first identified P-element insertions and then compared the frequency increases of the P-elements to the one of selected alleles.

The raw reads of evolved replicates at generation 60 were separately mapped to a TE-merged reference genome using bwa [66] version 0.7.9a (*bwasw* algorithm). The reference genome consists of the repeat-masked reference genome and TE sequences as described in [53]. The paired reads were restored (function *se2pe*), and a ppileup file was generated using PoPoolationTE2 [67]. All evolved replicates were down-sampled to 30, i.e., the coverage that 95% of sites are maintained (function *subsamplePpileup*), and TE insertions were identified with PoPoolationTE2 (functions *identifySignatures* and *frequency*). The identified TE insertions were filtered (*filterSignatures* with parameters—*max-otherte-count 2—max-structvar-count 2*), and the final set of insertions was identified (function *pairupSignatures*).

We used different frequency thresholds for identification of selected alleles (methods 1, 3, and 4). We filtered the identified P-elements by selecting those with frequencies ≥threshold that have insertion sites in the genomic regions corresponding to selected alleles. Furthermore, the P-elements with frequency increase must be present in at least one of the replicates that have frequency increase of ≥threshold for any given selected allele. Then, for each selected allele with an available P-element in the filtered data set, we computed delta (absolute frequency difference) using the frequency of the allele in any of the two replicates with highest frequency at generation 60 and the frequency of the P-element in those replicates.

### GO and pathway enrichment analyses

We used Gowinda [68] to associate the characteristic SNPs in the selected alleles with their biological functions and determine enrichment of any specific GO category. This tool corrects for biases introduced by gene lengths. The GO enrichment analysis was performed in gene mode, with 100,000 simulations. The associated GO terms were downloaded from GoMiner [69]. We repeated the enrichment analysis using pathways obtained from KEGG [70].

### Phenotypic assays

All phenotypic assays were performed in a common garden setting (with temperature fluctuating between 18°C and 28°C) to eliminate the possibility that uncontrolled environmental variation is affecting the phenotypic measurements. We reconstituted the ancestral population from the isofemale lines that were used to seed the original ancestral population. Because *N*_e_ of isofemale lines is very small, very limited adaptation is expected to have occurred during their maintenance in the lab [28,71]. Even mutations accumulated during the maintenance of the isofemale lines are not expected to have a major influence because they will be specific to individual isofemale lines and will thus represent only a small fraction in the reconstituted ancestral population. We refer to the reconstituted ancestral population as “ancestral population” hereafter. All 10 evolved replicates and the ancestral population were maintained for at least two generations at the assaying conditions with controlled density (400 eggs per bottle) prior to assays to avoid maternal effects.

#### A. Fecundity assay

At generation 103, between three to six subreplicates were set up for each of the 10 evolved replicates to ensure reliable fecundity estimates. Ten subreplicates were set up for the ancestral population. Immediately after eclosion, around 250 flies (females and males) were put in a bottle. Because the flies were collected under CO_2_ anesthesia, we only started measuring fecundity in two-day-old flies. For the next four days, which corresponds to the egg-laying period during the experimental evolution, the flies were daily transferred to new bottles without CO_2_ anesthesia, and the eggs in each bottle were counted. After the fourth day, females and males were separated, and the females were counted, dried, and weighed. Fecundity—measured as the total number of eggs laid per female during four days (between day two to five after eclosion)—was log10-transformed and analyzed using a linear model.

To test for the significance between fecundity of the ancestral and evolved (all 10 evolved replicates combined) populations, we initially fitted a linear mixed model with a fixed categorical effect (population) with two levels (ancestral and evolved) and a fixed continuous effect (average body weight). The subreplicates of the ancestral and evolved populations were included as a random effect. The mixed model was not significantly different from the linear fixed effects model tested using the function *anova*. Therefore, following the principle of parsimony, we present results from the linear fixed effects model (Fecundity_ijk_ ~ μ + population_i_ + average body weight_j_ + error_ijk_). The data met the assumptions of normality of the residuals and homogeneity of variance.

The model to test for differences between the 10 evolved replicates was the same as above, but with population being a fixed categorical effect with 10 levels. The mixed effects model including a random effect to model the covariance between the subreplicates of each evolved replicate was not significantly better than the fixed effects model and hence we dropped it in favor of the simpler fixed effects model. Significance of the fixed effects was tested using ANOVA F-tests. We present effect sizes as lsmeans calculated with package *lsmeans* [72] and used Tukey’s HSD to correct for multiple testing.

#### B. Resting metabolism assay

The evolved populations were at generation 113 when phenotyped for resting metabolic rate. The flies used for measurement of metabolic rate were maintained at controlled density as described above (evolved replicates for seven to nine and ancestral population for four to seven generations). Flies were collected immediately after eclosion and mated for 24 hours, and then females and males were separated and maintained in bottles with *Drosophila* medium (150 flies in a bottle). After 48 hours of CO_2_ anesthesia recovery, flies were used for metabolic rate measurement. Females and males were four to five and six to seven days old, respectively, during resting metabolic measurements. Our preliminary assays identified no significant difference in the metabolic rate of four- to five- and six- to seven-day-old males when groups of 25 males were tested in RC respirometry chambers (30 mL; Sable Systems, Las Vegas, Nevada). For measuring metabolic rate, 150 flies were transferred to a 250-mL bottle without CO_2_ anesthesia. To avoid desiccation and starvation, 50 mL of *Drosophila* medium was placed in each bottle and sealed with a stopper. The resting metabolic rate was measured by repeated CO_2_ emission measurements of stop-flow respirometry (Sable Systems). Flies of different replicates were randomly assigned to each bottle, and one bottle with only *Drosophila* medium was used as an empty control in each run. CO_2_ measurement was conducted at 18°C in the dark, overnight for at least 12 hours. During the measurement assay, an 8-channel multiplexer (RM8 Intelligent Multiplexer) controlled the sequential flushing and closing of eight bottles. Each bottle was flushed for 15 minutes at a constant flow rate of 75 μL/min. After the flush phase, the bottle was closed while the rest of bottles continued to be flushed. Therefore, CO_2_ in each bottle was measured every two hours. The flushed air passed through a magnesium perchlorate column to remove water, and CO_2_ was measured with a CA-10A carbon dioxide analyzer (Sable Systems). At the end of each run, the flies were counted, dried, and weighed. An in-house macro was used for computing the total CO_2_ emission during each flushing time and the mean flow rate by ExpeData software. The resting metabolic rate for each bottle was computed as the average of the three lowest data points [73]. The metabolic rate is presented as V_CO2_ μL h^−1^ mg^−1^. A total of 32 subreplicates (16 for each sex) were measured for the ancestral population. At least six subreplicates were measured for each evolved replicate (three for each sex).

To test for the significance between the metabolic rate of the ancestral and evolved (all 10 evolved replicates combined) populations, we initially fitted a linear mixed model with two fixed categorical effects (population and sex) each with two levels (population: ancestral and evolved; sex: female and male) and interaction between the fixed categorical effects. Subreplicates of the ancestral population and subreplicates of the evolved replicates were included as a random effect. Similar to fecundity, the mixed model was not significantly different from the linear fixed effects model. Therefore, we present results from the linear fixed-effects model (Metabolism_ijk_ ~ μ + population_i_ + sex_j_ + population_i_: sex_j_ + error_ijk_). The data met the assumptions of normality of the residuals and homogeneity of variance.

We used the same model as above to test for differences between the 10 evolved replicates, but with population being a fixed categorical effect with 10 levels. We included a random effect to model covariance between subreplicates of each evolved replicate, but it was not significantly better than the fixed effects model and was therefore dropped in favor of the simpler fixed effects model. Significance of the fixed effects was tested using ANOVA F-tests. We present effect sizes as lsmeans and used Tukey’s HSD to correct for multiple testing.

#### C. Body fat assay

We assayed the ancestral population and evolved replicates for fat content at generation 124. Similar to other phenotypic assays, the evolved replicates and ancestral population were maintained in a density-controlled common garden setting (for three to six generations). After eclosion, flies were mated for 1 day, and females and males were separated and placed in vials (eight flies in each vial). After 48 hours, the body fat was measured in four-day-old flies three hours after the start of the daily 28°C cycle. Homogenates were prepared as described in [74], and lipid measurements were performed by coupled colorimetric assay as described in [75]. Flies were placed in a 2-mL screwcap tube containing 600 μL 0.05% Tween-20 and two 5-mm sterile metal beads and were homogenized by a SPEX SamplePrep 1600 MiniG for 2 minutes at 1,475 rpm. Homogenates were heat-inactivated for 5 minutes at 70°C and centrifuged for 9 minutes at 9,000 rpm, and 200 μL of the supernatant was transferred to an Eppendorf tube and immediately used for lipid measurements. We used a Glycerol standard (Sigma G7793) as reference. The amount of 50 μL of supernatant and standards (1.2, 1, 0.8, 0.6, 0.4, 0.2, 0.1, 0 mg/mL) were transferred to a 96-well plate, and blank absorbance was measured at 540 nm in an EnSpire 2300 Microplate Reader (PerkinElmer). The amount of 200 μL of Triglyceride Working Reagent (Sigma, Catalog number TR0100) was added to each sample and standards and was incubated at 37°C for 30 minutes with mild shaking. After the incubation time, the final absorbance was measured at 540 nm. The two measurements were first blank-corrected by subtraction of blank (i.e., 0.05% Tween-20), and then the second absorbance was subtracted from the first absorbance. Glycerol standards were done in duplicates, and two measurements were averaged for making the standard curves with polynomial regression line to compute the concentration of triglyceride (TAG) in the samples. Fat content is expressed as μg TAG equivalents per fly. A total of 26 subreplicates (13 for each sex) were measured for the ancestral population, and eight subreplicates were measured for each evolved replicate (four for each sex).

The measured fat content (μg TAG equivalents/fly) was log-transformed and analyzed using a linear model. To test for the significance between the fat content of the ancestral and evolved (all 10 evolved replicates combined) populations, we initially fitted a linear mixed model with two fixed categorical effects (population and sex) each with two levels (population: ancestral and evolved; sex: female and male), a fixed continuous effect (average body weight), and interaction between the fixed categorical effects. The subreplicates of the ancestral population and subreplicates of the evolved replicates were included as a random effect. Due to error during weighing, for six subreplicates, we did not provide weight values in the linear model. The average body weight was not significant and was therefore dropped from the model. The mixed model was also not significantly different from the linear fixed effects model. Therefore, we present results from the linear fixed effects model (Lipid_ijk_ ~ μ + population_i_ + sex_j_ + population_i_: sex_j_ + error_ijk_). The data met the assumptions of normality of the residuals and homogeneity of variance.

The same model as above was used to test for differences between the 10 evolved replicates. Here, we treated population as a fixed categorical effect with 10 levels. Similar to other phenotypic assays, the mixed effects model including a random effect was not significantly better than the fixed effects model and was therefore dropped in favor of the simpler fixed effects model. The interaction between fixed effects was dropped from the final model because it was not significant. Significance of the fixed effects was tested using ANOVA F-tests. We present effect sizes as lsmeans and used Tukey’s HSD to correct for multiple testing.

All the analysis and data visualization were performed in Python version 2.7.10 (Python Language Reference; Python Software Foundation) and R version 3.3.1 (R development Core Team; 2015).

## Supporting information

S1 Fig Increased fitness and phenotypic similarity among 10 evolved replicates. (A) Evolved females are more fecund than the ancestral population (ANCOVA, Tukey’s HSD test *p* < 0.0001). The number of eggs laid over four days (two to five days after eclosion) were counted, (B) Females of 10 evolved replicates are equally fecund (ANCOVA, Tukey’s HSD test, *p* > 0.05). Similar fat content (C) and metabolic rate (D) were measured among males of the evolved replicates (two-way ANOVA, Tukey’s HSD test *p* > 0.05). The bars show least-squares means of the linear model, and error bars depict 95% confidence levels of least-squares means. The dark khaki horizontal bar shows the 95% confidence levels of least-squares means of the ancestral population. Data deposited in the Dryad Repository: https://doi.org/10.5061/dryad.rr137kn. HSD, honest significant difference; TAG, triglyceride.
(PDF)

S2 Fig Size distribution of the reconstructed haplotype blocks in evolved replicates. Fifty percent of the haplotype blocks were smaller than 100 Kb, but approximately 25% were larger than 1 Mb. Data available in S1 Table.
(PNG)

S3 Fig Genomic heterogeneity in evolved replicates. The RFS shows the frequency distribution of replicates in which selected alleles increase in frequency. Different thresholds were used to identify an allele as selected in each replicate; Top panel: ≥0.1 (method 1) and ≥0.2 AFC (method 2): an allele with ≥0.1/0.2 frequency change, bottom panel: ≥5% (method 3) and ≥10% (method 4) ASFC: lower 5%/10% tail of AFC in selective sweep simulations (Materials and methods “Different approaches to determine the presence of selected alleles and their frequencies”). Regardless of the threshold used to determine a selected allele in a given replicate, a heterogeneous pattern among replicates is observed. Data deposited in the Dryad Repository: https://doi.org/10.5061/dryad.rr137kn. AFC, allele frequency change; ASFC, allele-specific frequency change; RFS, replicate frequency spectrum.
(PNG)

S4 Fig Different simulation scenarios used to contrast selective sweep and QT paradigms. We compare different adaptive sweep and QT scenarios to the empirical data: selective sweep simulations of alleles without (panel A) and with (panel B) linkage were studied, as well as different aspects of a QT paradigm: genetic redundancy (panel C) and simulations of AFCs assuming a QT with stabilizing selection without (panel D) and with (panel E) linkage among alleles. Sweep simulations (panel A and B) were performed for 99 (A1–6, B1–6) and 79 alleles (increasing in more than four replicates, A7 and B7). The selection coefficient (*s*) was estimated using the median frequency trajectories of selected alleles in replicates with ≥0.1 (method 1, orange circles) and ≥0.2 (method 2, brown circles) AFC. *s* was also estimated using the median frequency change in replicates with ≥5% (method 3, light blue circle) and ≥10% (method 4, dark blue circle) ASFC. *s* and starting frequency of the selected alleles were estimated using either all SNP characteristic of a given selected allele (“full alleles”) or only the “core region” (methods 5 and 6). See Materials and methods “Different approaches to determine the presence of selected alleles and their frequencies” for description of different methods and the definition of core region. The details of the redundancy paradigm are explained in Materials and methods “C. Genetic redundancy paradigm.” Simulations of a QT with stabilizing selection were performed with 99 loci using starting frequency of selected alleles (“full allele”) and equal effect sizes of all alleles using unlinked (panel D) or linked (panel E) alleles. AFC, allele frequency change; ASFC, allele-specific frequency change; QT, quantitative trait; SNP, single nucleotide polymorphism.
(PDF)

S5 Fig Selection coefficients (*s*) of selected alleles using different approaches to estimate the frequency of a given selected allele. The median frequency of each allele (the median frequency of all marker SNPs of a selected allele) was computed, and the frequency trajectory of replicates with ≥0.1 (method 1) and ≥0.2 (method 2) AFC until generation 60 were used for *s* estimation (A–C). ASFC thresholds of ≥5% (method 3) and ≥10% (method 4) were used to determine selected alleles in each replicate, *s* in replicates with selected alleles was estimated and median is reported (E and H). Panels K and N show the estimated *s* for the region with the highest estimated *s* in each allele (methods 5 and 6). See Materials and methods “Different approaches to determine the presence of selected alleles and their frequencies” for description of different methods and definition of core region). *s* was estimated for replicates with a selected allele using different frequency increase thresholds (methods 2–6) and the median *s* across the replicates is reported in B, E, H, K, and N (method 1 is in Fig 3B), whereas in A, C, F, I, L, and O, the calculated *s* for all the replicates with frequency change more than specified threshold is reported. The starting frequency of alleles with ≥0.2 AFC (D), ≥5% ASFC (G), ≥10% ASFC (J), and ≥0.1 AFC (M) and ≥0.2 AFC (P) for the core regions of selected alleles is shown. The estimated *s* using all approaches agrees (similar mean and median), but frequency trajectories of replicates with ≥0.1 AFC (Fig 3B) and ≥5% ASFC (E) resulted in more conservative *s* estimates. Data deposited in the Dryad Repository: https://doi.org/10.5061/dryad.rr137kn. AFC, allele frequency change; ASFC, allele-specific frequency change; QT, quantitative trait.
(PDF)

S6 Fig Genomic heterogeneity of simulations based on a selective sweep paradigm with a constant *s* across replicates and no linkage. The RFS shows the frequency distribution of replicates in which selected alleles increase in frequency. The RFS of experimental data (observed) is indicated by salmon dots. The expected distribution of RFS was obtained by computer simulations (Materials and methods “A. Sweep paradigm with a constant *s* across replicates and no linkage”) and is indicated in light gray (mean: black line). Simulations performed with median *s* estimated from frequency trajectories of replicates with (A) ≥0.2 AFC for an allele (S5B Fig), (B) ≥5% allele-specific frequency change, i.e., ASFC (S5E Fig), and (C) ≥10% ASFC (S5H Fig). (D, E) Simulations performed with *s* estimated for the core region of each selected allele using frequency trajectories of replicates with ≥0.1 (D, S5K Fig) and ≥0.2 AFC (E, S5N Fig). (F) Simulations performed using estimated *s* for alleles that increased in frequency (≥0.1) in ≥4 replicates in experimental data. Note that alleles identified in only 1–3 replicates had high *s* and low starting frequency (S10 Fig) and were therefore excluded from these simulations. Starting frequencies of simulated alleles match the empirical data (A: S5D Fig; B: S5G Fig; C: S5J Fig; D: S5M Fig; E: S5P Fig; F: Fig 3A). All simulations assume free recombination among loci. The difference between the empirical and simulated data is shown as Σ(obs − exp)^2^. Data deposited in the Dryad Repository: https://doi.org/10.5061/dryad.rr137kn. AFC, allele frequency change; ASFC, allele-specific frequency change; RFS, replicate frequency spectrum.
(PDF)

S7 Fig Genomic heterogeneity of simulations based on a sweep paradigm with linkage and a constant *s* across replicates. RFS shows the frequency distribution of replicates in which selected alleles increase in frequency. RFS of experimental data (observed) is indicated by salmon dots. The expected distribution of RFS was obtained by computer simulations (see Materials and methods “B. Sweep paradigm with linkage and a constant *s* across replicates”) and is indicated in dark gray (mean in black line). The same selection coefficients and starting frequencies were used as in S6 Fig. Data deposited in the Dryad Repository: https://doi.org/10.5061/dryad.rr137kn. RFS, replicate frequency spectrum.
(PDF)

S8 Fig **Comparison of the genetic heterogeneity (A–B) and replicate similarity (C–D) of the selective sweep and QT paradigm simulations to the observed data.** (A, B) The difference between RFS of empirical (observed) and the simulated (expected) data. For 1,000 iterations of each simulation, the difference between empirical and simulated RFS, Σ(obs − exp)^2^, is shown. (C, D) Pairwise Jaccard indices among 10 replicates in empirical data and in 1,000 iterations of each simulation. The threshold to determine selected alleles in empirical and simulated data in each replicate is ≥5% ASFC in A, C and ≥10% ASFC in B, D (methods 3 and 4 in Materials and methods “Different approaches to determine the presence of selected alleles and their frequencies”). Data in panels A and C were simulated under QT paradigm without (S11A Fig) and with (S12A Fig) linkage, redundancy (S9A Fig) and sweep paradigm without (S6B Fig) and with (S7B Fig) linkage. Data in panels B and D were simulated under QT paradigm without (S11B Fig) and with (S12B Fig) linkage, redundancy (S9B Fig) and sweep paradigm without (S6C Fig) and with (S7C Fig) linkage. Σ(obs − exp)^2^ and Jaccard indices across simulations are compared using one-way ANOVA, Tukey’s HSD test, *p* < 10^−5^. Data deposited in the Dryad Repository: https://doi.org/10.5061/dryad.rr137kn. AFC, allele frequency change; ASFC, allele-specific frequency change; HSD, honest significant difference; RFS, replicate frequency spectrum; QT, quantitative trait.
(PDF)

S9 Fig Genomic heterogeneity of simulations based on the redundancy paradigm. The RFS shows the frequency distribution of replicates in which selected alleles increase in frequency (threshold; A: ≥5% ASFC [method 3 in Materials and methods “Different approaches to determine the presence of selected alleles and their frequencies”]; B: ≥10% ASFC [method 4]). The RFS of experimental data (observed) is indicated by salmon dots. The expected distribution of RFS was obtained by 1,000 iterations of delete-d jackknifing computer simulations (Materials and methods “C. Genetic redundancy paradigm”) and is indicated in blue (mean: black line). The number of randomly drawn alleles from 99 alleles in each set of simulations was equal to the number of selected alleles in each replicate with ≥5% and ≥10% ASFC in A and B, respectively. Data deposited in the Dryad Repository: https://doi.org/10.5061/dryad.rr137kn. ASFC, allele-specific frequency change; RFS, replicate frequency spectrum.
(PDF)

S10 Fig Characteristics of selected alleles. Starting frequency (top panel) and selection coefficient (bottom panel) of the selected alleles classified by the number of replicates in which a given selected allele has ≥0.1 frequency increase at generation 60 (method 1 in Materials and methods “Different approaches to determine the presence of selected alleles and their frequencies”). The selected alleles that increased in frequency (≥0.1) in only one to three replicates have low starting frequencies and the highest estimated *s*. Boxplots show the first and third quartile of the distribution, and horizontal bars in each box shows the median in each category. The data of individual selected alleles are shown as scattered dots in each boxplot. Data deposited in the Dryad Repository: https://doi.org/10.5061/dryad.rr137kn.
(PDF)

S11 Fig Genomic heterogeneity of simulations based on the QT paradigm without linkage among alleles. The RFS shows the frequency distribution of replicates in which selected alleles increase in frequency (threshold; A: ≥5% ASFC [method 3 in Materials and methods “Different approaches to determine the presence of selected alleles and their frequencies”]; B: ≥10% ASFC [method 4]). The RFS of experimental data (observed) is indicated by salmon dots. The expected distribution of RFS was obtained by 1,000 iterations of computer simulations (Materials and methods “D. QT paradigm without linkage”) in 10 replicates for 60 generations with 99 contributing alleles having the same starting frequency as the selected alleles in the empirical data (Fig 3A) and is indicated in light green (mean in black line). Data deposited in the Dryad Repository: https://doi.org/10.5061/dryad.rr137kn. ASFC, allele-specific frequency change; QT, quantitative trait; RFS, replicate frequency spectrum.
(PDF)

S12 Fig Genomic heterogeneity of simulations based on the QT paradigm with linkage among alleles. RFS shows the frequency distribution of replicates in which selected alleles increase in frequency (threshold; A: ≥5% ASFC [method 3 in Materials and methods “Different approaches to determine the presence of selected alleles and their frequencies”]; B: ≥10% ASFC [method 4]). The RFS of experimental data (observed) is indicated by salmon dots. The expected distribution of RFS was obtained by 1,000 iterations of computer simulations (Materials and methods: “E. QT paradigm with linkage”) in 10 replicates for 60 generations with 99 contributing alleles having the same starting frequency as the selected alleles in the empirical data (Fig 3A) and is indicated in dark green (mean in black line). Data deposited in the Dryad Repository: https://doi.org/10.5061/dryad.rr137kn. ASFC, allele-specific frequency change; QT, quantitative trait; RFS, replicate frequency spectrum.
(PDF)

S13 Fig Fitness functions used for the simulation of QT paradigm. (A) Gaussian fitness function used in QT paradigm without linkage (D in S4 Fig) optimum phenotype = 0.6, standard deviation = 0.3, and fitness range from 0.5 to 4.5. (B) Gaussian fitness function used in QT paradigm with linkage (E in S4 Fig) optimum phenotype = −1.3, standard deviation = 1.2, and fitness range from 0.5 to 4.5. QT, quantitative trait.
(PDF)

S1 Table Characteristics of the reconstructed haplotype blocks. In cases in which the block spans both arms of the chromosome, both chromosome arms are specified. chr, chromosome; num, an arbitrary number given to the haplotype block of each chromosome; pos (bp), the genomic position of block in the chromosome; size (kb), length of the block in kb; SNP nums, the number of SNP in the haplotype block; rising reps. num., the number of replicates in which a haplotype block has at least 0.1 frequency increase; rising reps., the replicates with at least 0.1 frequency increase; columns rep1 to rep10 show the number of haplotypes that share at least 80% of the SNPs characteristic to the haplotype block in replicates 1, 3, 4, 7, and 10, respectively, for which evolved haplotypes were sequenced. The number of available haplotypes is given in parentheses; validated haplos. num. evolved, total number of haplotypes across all five replicates in which the haplotype block is validated by at least 80% of the SNPs characteristic to the block; validated haplos. num. ancestral, total number of haplotypes from 189 ancestral haplotypes in which the haplotype block is validated by at least 80% of the SNPs characteristic to the block. SNP, single nucleotide polymorphism.
(XLSX)

S2 Table Enrichment of gene functions in selected alleles. “*” indicates uncorrected for multiple testing. “$” indicates *p*-value after adjustment for multiple testing.
(XLSX)

S3 Table Enrichment of KEGG pathways in selected alleles. “*” indicates uncorrected for multiple testing. “$” indicates *p*-value after adjustment for multiple testing.
(XLSX)

S4 Table Absolute frequency difference of the identified P-elements (in selected alleles) and selected alleles. Chr.: left and right arms of chromosomes are concatenated as some haplotype blocks span the centromere. No.: an arbitrary number given to the haplotype block of each chromosome (similar to the numbers in S1 Table). Delta: the absolute frequency difference between the frequency of the allele in any of the two replicates with highest frequency at generation 60 and the frequency of the P-element in those replicates. Thresh0.1: frequency change of ≥0.1 after 60 generations (method 1 in Materials and methods “Different approaches to determine the presence of selected alleles and their frequencies”), Thresh5%: 5% ASFC (method 3), Thresh10%: 10% ASFC (method 4). *Using thresh0.1, the replicate with the highest allele frequency for this haplotype block did not have P-element insertion, thus the replicate with the second highest allele frequency was tested, whereas using threshold 5% and 10%, the replicate with the highest allele frequency for this haplotype block had P-element insertion. ASFC, allele-specific frequency change.
(XLSX)

S5 Table Estimated *N_e_* in evolved replicates for autosomes and the X chromosome. *N*_*e*_, effective population size.
(XLSX)

S6 Table Summary of regression models to identify factors affecting the estimated selection coefficient (*s*). (A) The size of haplotype block is used as physical or genetic distance. (B) The estimated *s* was computed based on the frequency trajectory of a selected allele in replicates with ≥5% and ≥10% ASFC (methods 3 and 4 in Materials and methods “Different approaches to determine the presence of selected alleles and their frequencies”). Standard errors are shown in parentheses. The table has been made using stargazer (Hlavac, Marek (2018). stargazer: Well-Formatted Regression and Summary Statistics Tables. R package version 5.2.2. https://CRAN.R-project.org/package=stargazer). ASFC, allele-specific frequency change.
(DOCX)

S7 Table **Details of DNA extraction and library preparation for Pool-Seq (A) and haplotype samples (B).** (A) For the founder population, nomenclature is as follows: species_population_selectionRegime_replicate (e.g., Dsim_Fl_Base_1), and for the evolved populations, nomenclature is as follows: species_population_selectionRegime_generation_replicate (e.g., Dsim_Fl_Hot_F10_1). (B) Nomenclature of the samples is as follows: species_population_selectionRegime_generation_replicate_cross (e.g., Dsim_Fl_Hot_F88_r1_c1).
(XLSX)

S8 Table The coverage of SNPs for all time points and replicates. (XLSX)

## Abbreviations

AFC: allele frequency change
CMH: Cochran-Mantel-Haenszel
E&R: evolve and resequence
GO: gene ontology
GWAS: genome-wide association study
HSD: honest significant difference
*N*_e_: effective population size
QT: quantitative trait; QTL, quantitative trait locus
RFS: replicate frequency spectrum
*s*: selection coefficient
SFS: site frequency spectrum
SNP: single nucleotide polymorphism
TAG: triglyceride
TE: transposable element
