# Supplementary figures and images for: Genetic redundancy fuels polygenic adaptation in Drosophila
Source: PLoS Biol. 2019 Feb 4;17(2):e3000128. doi: 10.1371/journal.pbio.3000128 (PMC6375663; doi:10.1371/journal.pbio.3000128)

**A**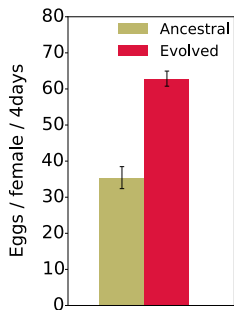**B**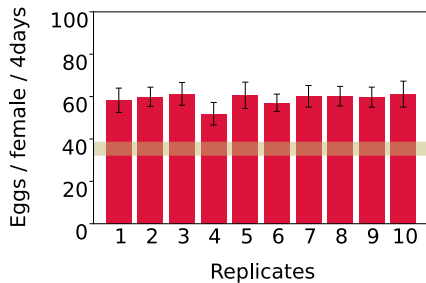**C**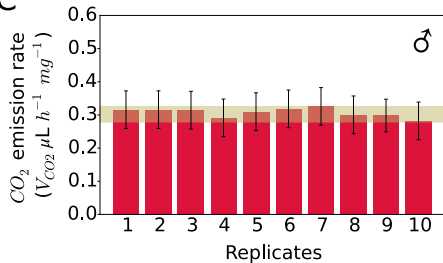**D**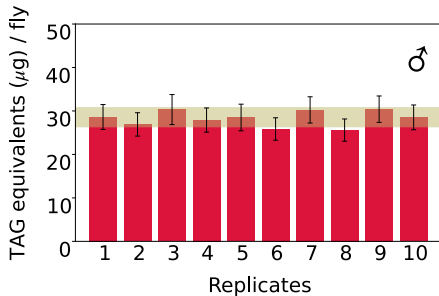

Supplement: S1 Fig — (A) Evolved females are more fecund than the ancestral population (ANCOVA, Tukey’s HSD test p < 0.0001). The number of eggs laid over four days (two to five days after eclosion) were counted, (B) Females of 10 evolved replicates are equally fecund (ANCOVA, Tukey’s HSD test, p > 0.05). Similar fat content (C) and metabolic rate (D) were measured among males of the evolved replicates (two-way ANOVA, Tukey’s HSD test p > 0.05). The bars show least-squares means of the linear model, and error bars depict 95% confidence levels of least-squares means. The dark khaki horizontal bar shows the 95% confidence levels of least-squares means of the ancestral population. Data deposited in the Dryad Repository: https://doi.org/10.5061/dryad.rr137kn. HSD, honest significant difference; TAG, triglyceride. (PDF) [file pbio.3000128.s001.pdf]

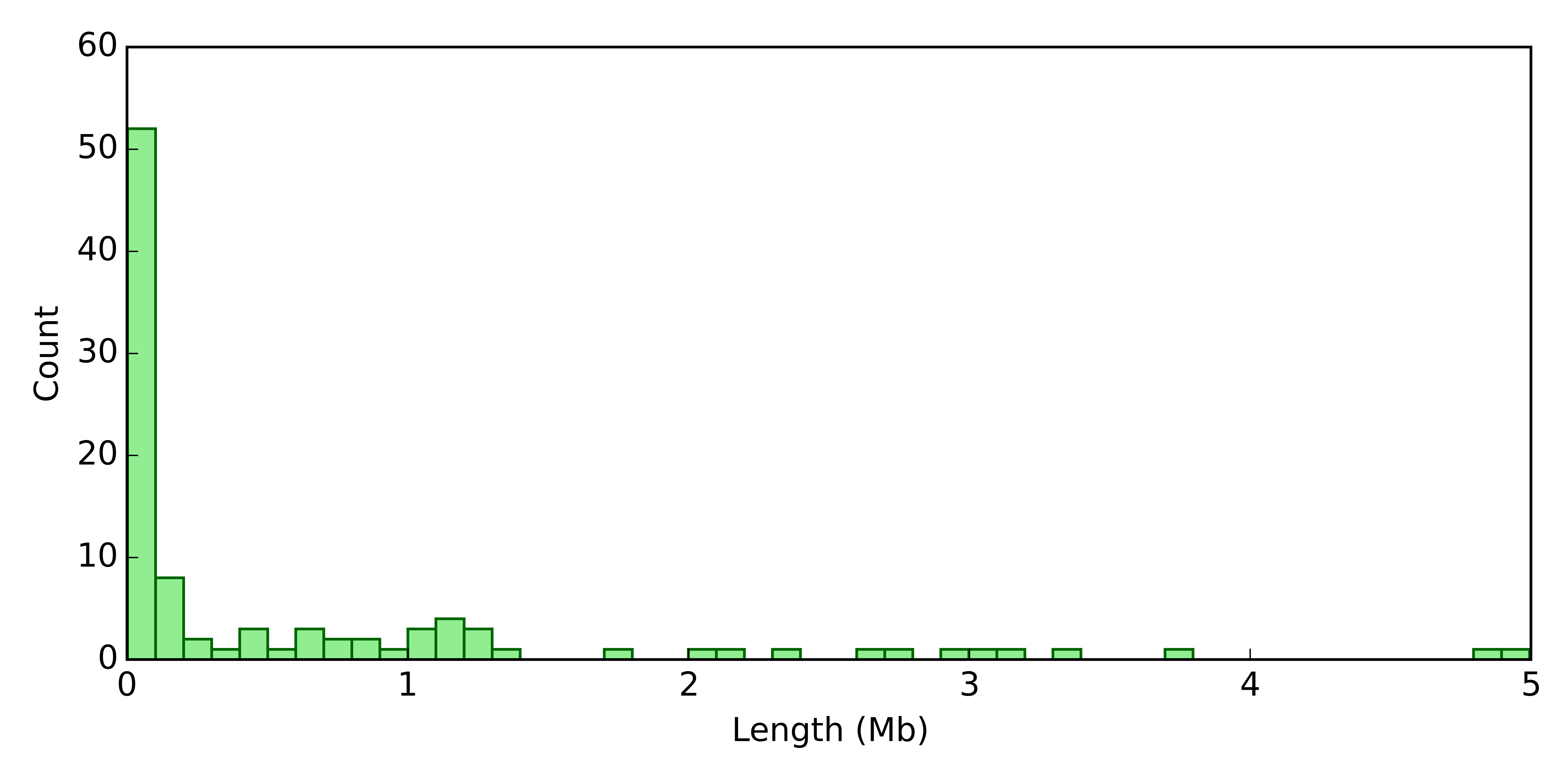

Supplement: S2 Fig — Fifty percent of the haplotype blocks were smaller than 100 Kb, but approximately 25% were larger than 1 Mb. Data available in S1 Table. (PNG) [file pbio.3000128.s002.png]

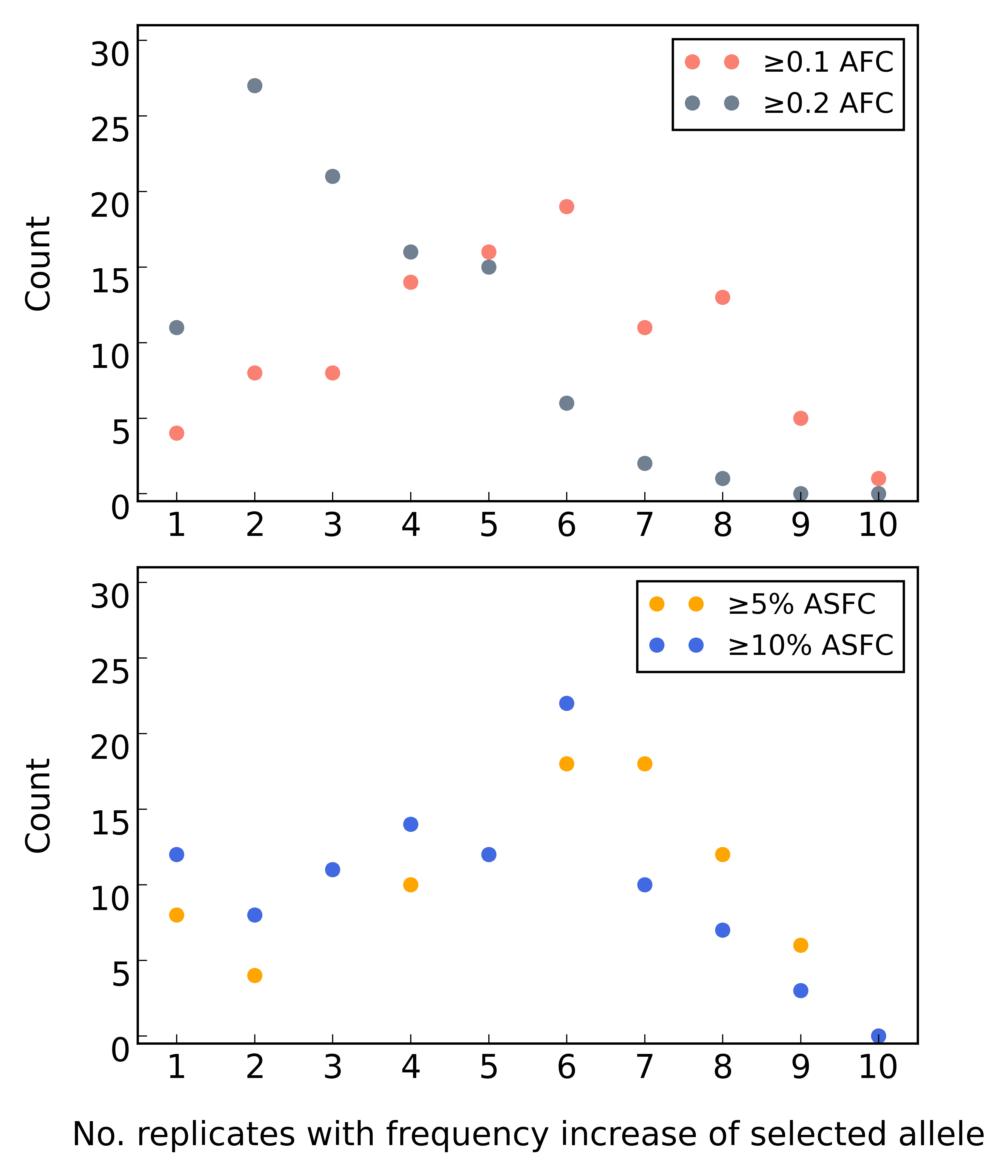

Supplement: S3 Fig — The RFS shows the frequency distribution of replicates in which selected alleles increase in frequency. Different thresholds were used to identify an allele as selected in each replicate; Top panel: ≥0.1 (method 1) and ≥0.2 AFC (method 2): an allele with ≥0.1/0.2 frequency change, bottom panel: ≥5% (method 3) and ≥10% (method 4) ASFC: lower 5%/10% tail of AFC in selective sweep simulations (Materials and methods “Different approaches to determine the presence of selected alleles and their frequencies”). Regardless of the threshold used to determine a selected allele in a given replicate, a heterogeneous pattern among replicates is observed. Data deposited in the Dryad Repository: https://doi.org/10.5061/dryad.rr137kn. AFC, allele frequency change; ASFC, allele-specific frequency change; RFS, replicate frequency spectrum. (PNG) [file pbio.3000128.s003.png]

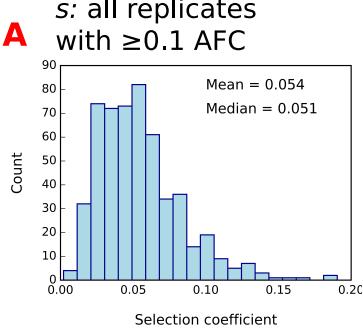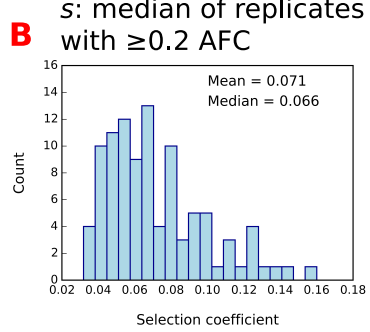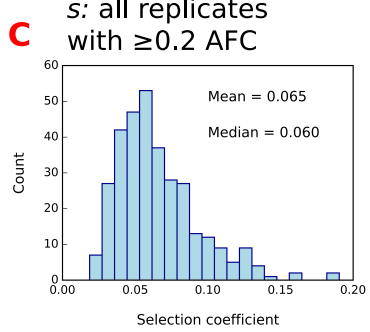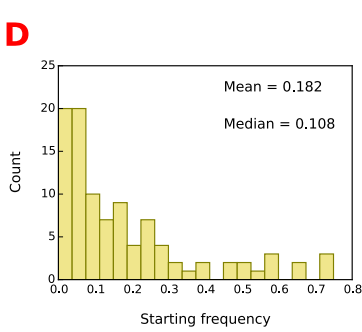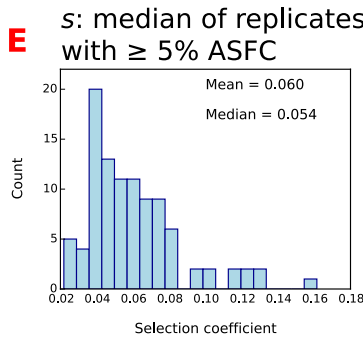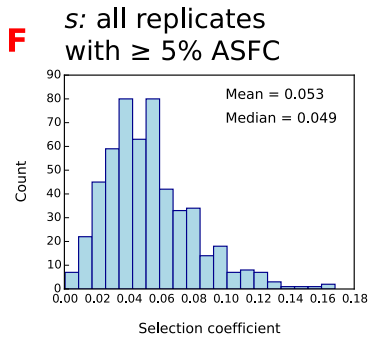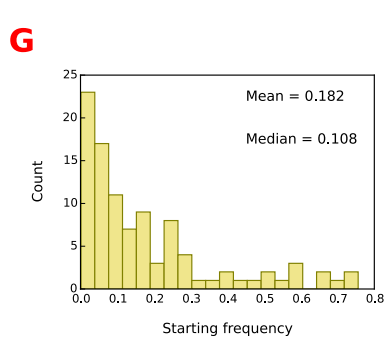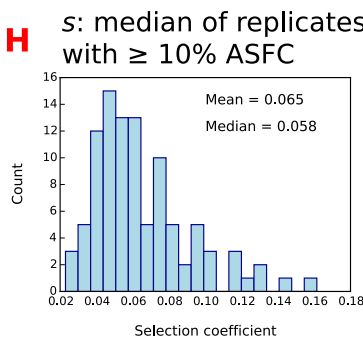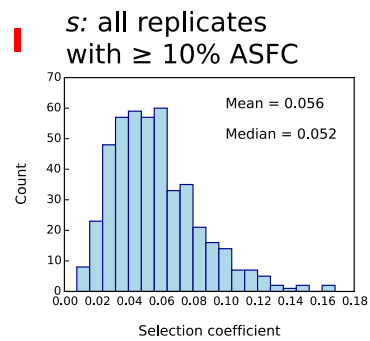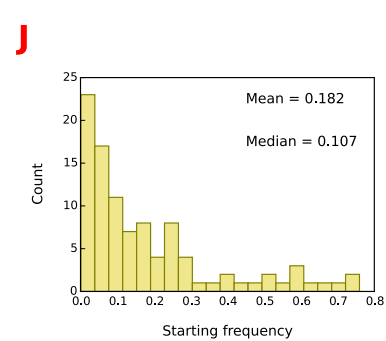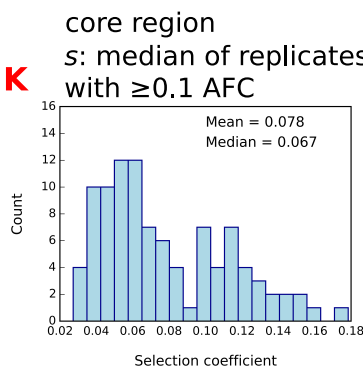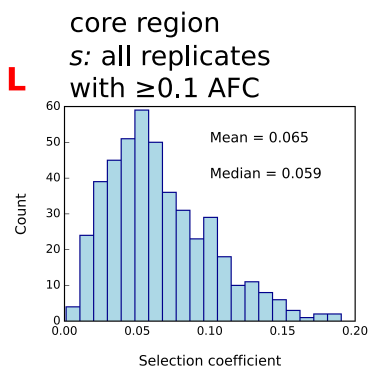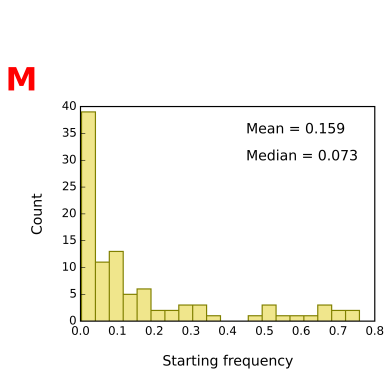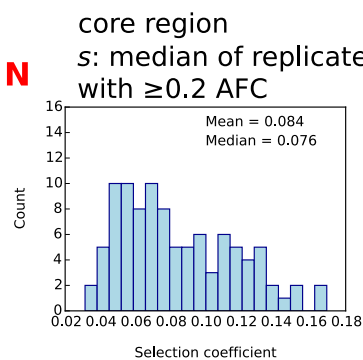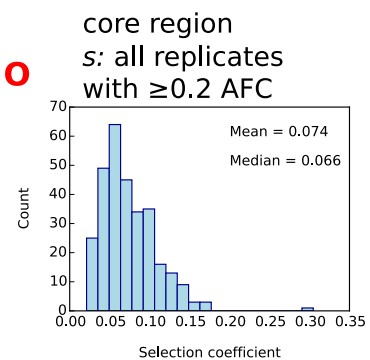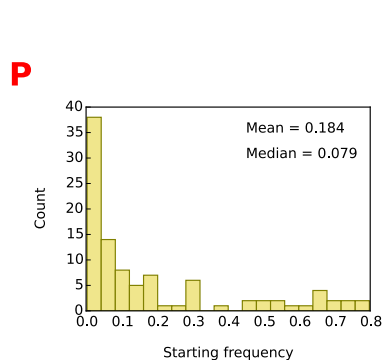

Supplement: S5 Fig — The median frequency of each allele (the median frequency of all marker SNPs of a selected allele) was computed, and the frequency trajectory of replicates with ≥0.1 (method 1) and ≥0.2 (method 2) AFC until generation 60 were used for s estimation (A–C). ASFC thresholds of ≥5% (method 3) and ≥10% (method 4) were used to determine selected alleles in each replicate, s in replicates with selected alleles was estimated and median is reported (E and H). Panels K and N show the estimated s for the region with the highest estimated s in each allele (methods 5 and 6). See Materials and methods “Different approaches to determine the presence of selected alleles and their frequencies” for description of different methods and definition of core region). s was estimated for replicates with a selected allele using different frequency increase thresholds (methods 2–6) and the median s across the replicates is reported in B, E, H, K, and N (method 1 is in Fig 3B), whereas in A, C, F, I, L, and O, the calculated s for all the replicates with frequency change more than specified threshold is reported. The starting frequency of alleles with ≥0.2 AFC (D), ≥5% ASFC (G), ≥10% ASFC (J), and ≥0.1 AFC (M) and ≥0.2 AFC (P) for the core regions of selected alleles is shown. The estimated s using all approaches agrees (similar mean and median), but frequency trajectories of replicates with ≥0.1 AFC (Fig 3B) and ≥5% ASFC (E) resulted in more conservative s estimates. Data deposited in the Dryad Repository: https://doi.org/10.5061/dryad.rr137kn. AFC, allele frequency change; ASFC, allele-specific frequency change; QT, quantitative trait. (PDF) [file pbio.3000128.s005.pdf]

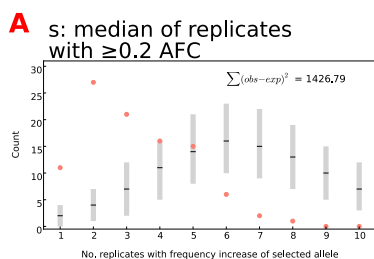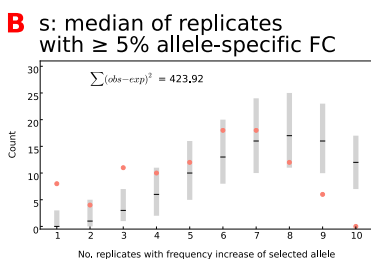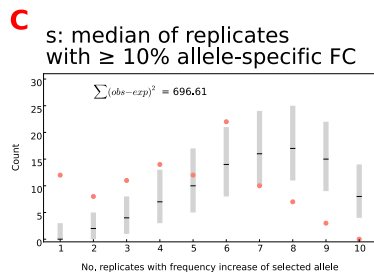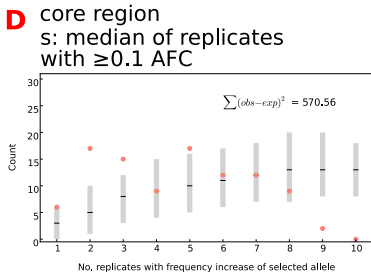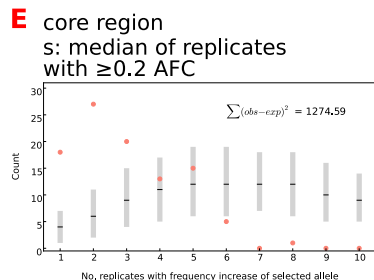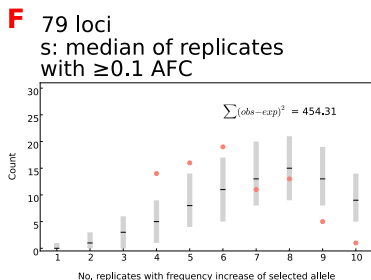

Supplement: S6 Fig — The RFS shows the frequency distribution of replicates in which selected alleles increase in frequency. The RFS of experimental data (observed) is indicated by salmon dots. The expected distribution of RFS was obtained by computer simulations (Materials and methods “A. Sweep paradigm with a constant s across replicates and no linkage”) and is indicated in light gray (mean: black line). Simulations performed with median s estimated from frequency trajectories of replicates with (A) ≥0.2 AFC for an allele (S5B Fig), (B) ≥5% allele-specific frequency change, i.e., ASFC (S5E Fig), and (C) ≥10% ASFC (S5H Fig). (D, E) Simulations performed with s estimated for the core region of each selected allele using frequency trajectories of replicates with ≥0.1 (D, S5K Fig) and ≥0.2 AFC (E, S5N Fig). (F) Simulations performed using estimated s for alleles that increased in frequency (≥0.1) in ≥4 replicates in experimental data. Note that alleles identified in only 1–3 replicates had high s and low starting frequency (S10 Fig) and were therefore excluded from these simulations. Starting frequencies of simulated alleles match the empirical data (A: S5D Fig; B: S5G Fig; C: S5J Fig; D: S5M Fig; E: S5P Fig; F: Fig 3A). All simulations assume free recombination among loci. The difference between the empirical and simulated data is shown as Σ(obs − exp)2. Data deposited in the Dryad Repository: https://doi.org/10.5061/dryad.rr137kn. AFC, allele frequency change; ASFC, allele-specific frequency change; RFS, replicate frequency spectrum. (PDF) [file pbio.3000128.s006.pdf]

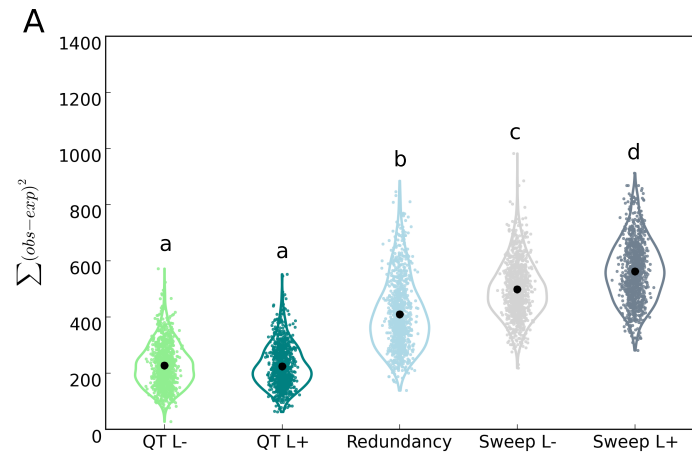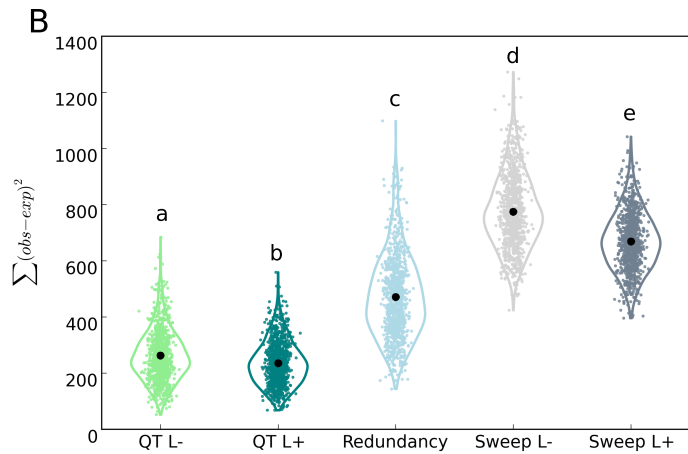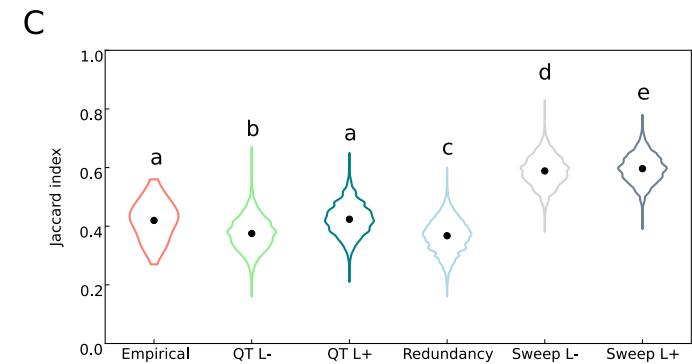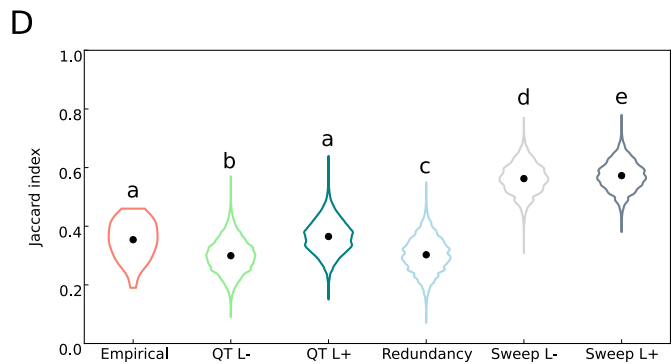

Supplement: S8 Fig — Comparison of the genetic heterogeneity (A–B) and replicate similarity (C–D) of the selective sweep and QT paradigm simulations to the observed data. (A, B) The difference between RFS of empirical (observed) and the simulated (expected) data. For 1,000 iterations of each simulation, the difference between empirical and simulated RFS, Σ(obs − exp)2, is shown. (C, D) Pairwise Jaccard indices among 10 replicates in empirical data and in 1,000 iterations of each simulation. The threshold to determine selected alleles in empirical and simulated data in each replicate is ≥5% ASFC in A, C and ≥10% ASFC in B, D (methods 3 and 4 in Materials and methods “Different approaches to determine the presence of selected alleles and their frequencies”). Data in panels A and C were simulated under QT paradigm without (S11A Fig) and with (S12A Fig) linkage, redundancy (S9A Fig) and sweep paradigm without (S6B Fig) and with (S7B Fig) linkage. Data in panels B and D were simulated under QT paradigm without (S11B Fig) and with (S12B Fig) linkage, redundancy (S9B Fig) and sweep paradigm without (S6C Fig) and with (S7C Fig) linkage. Σ(obs − exp)2 and Jaccard indices across simulations are compared using one-way ANOVA, Tukey’s HSD test, p < 10−5. Data deposited in the Dryad Repository: https://doi.org/10.5061/dryad.rr137kn. AFC, allele frequency change; ASFC, allele-specific frequency change; HSD, honest significant difference; RFS, replicate frequency spectrum; QT, quantitative trait. (PDF) [file pbio.3000128.s008.pdf]

A

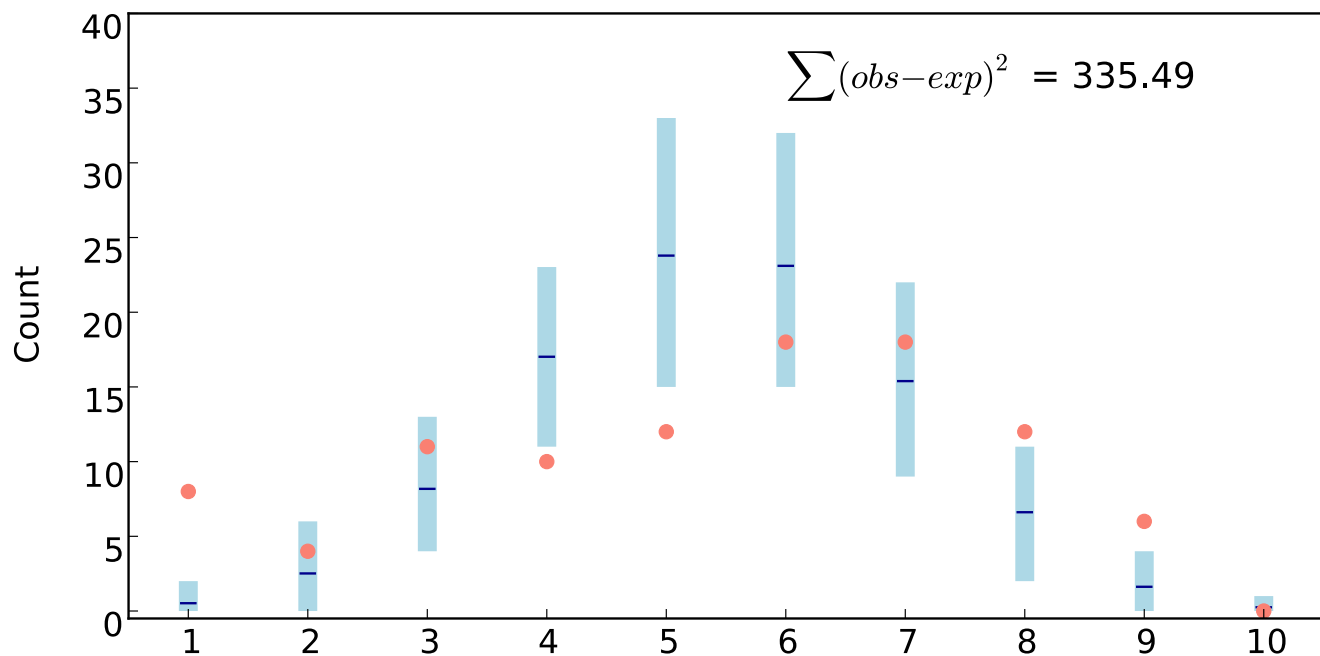

B

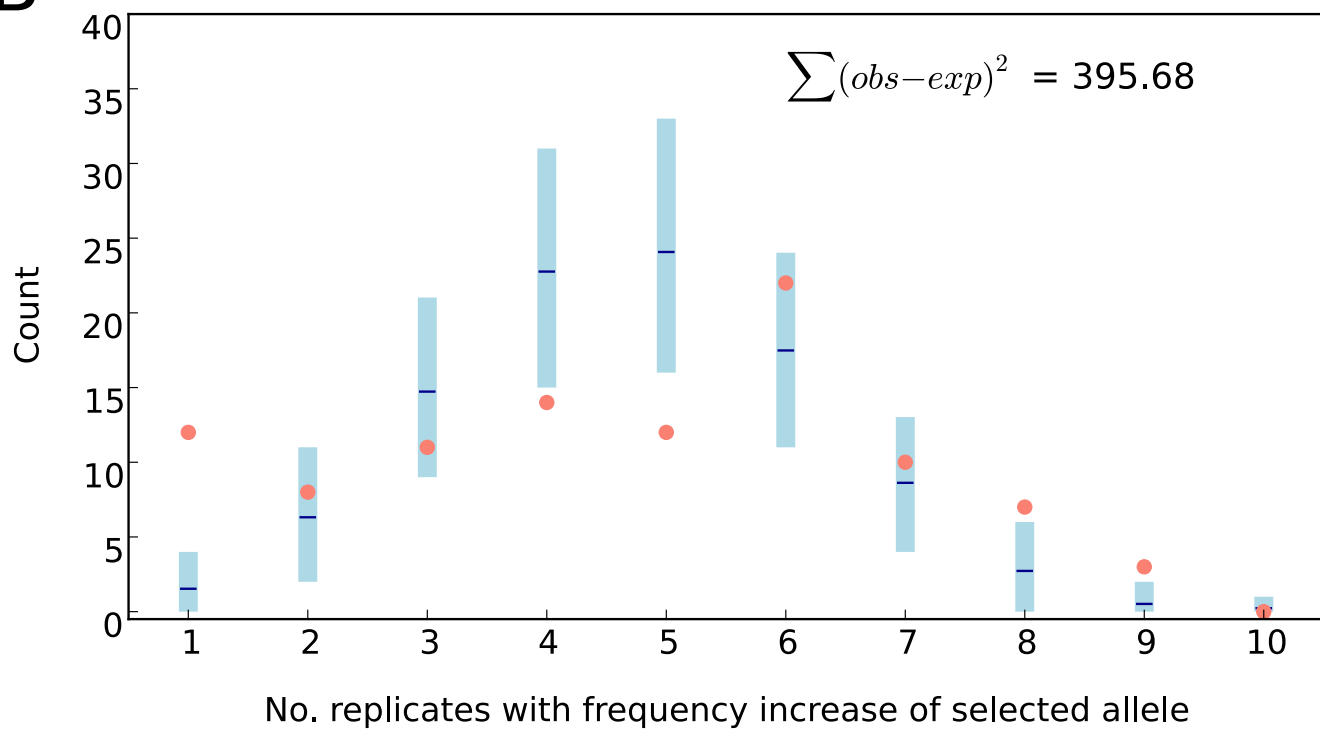

Supplement: S9 Fig — The RFS shows the frequency distribution of replicates in which selected alleles increase in frequency (threshold; A: ≥5% ASFC [method 3 in Materials and methods “Different approaches to determine the presence of selected alleles and their frequencies”]; B: ≥10% ASFC [method 4]). The RFS of experimental data (observed) is indicated by salmon dots. The expected distribution of RFS was obtained by 1,000 iterations of delete-d jackknifing computer simulations (Materials and methods “C. Genetic redundancy paradigm”) and is indicated in blue (mean: black line). The number of randomly drawn alleles from 99 alleles in each set of simulations was equal to the number of selected alleles in each replicate with ≥5% and ≥10% ASFC in A and B, respectively. Data deposited in the Dryad Repository: https://doi.org/10.5061/dryad.rr137kn. ASFC, allele-specific frequency change; RFS, replicate frequency spectrum. (PDF) [file pbio.3000128.s009.pdf]

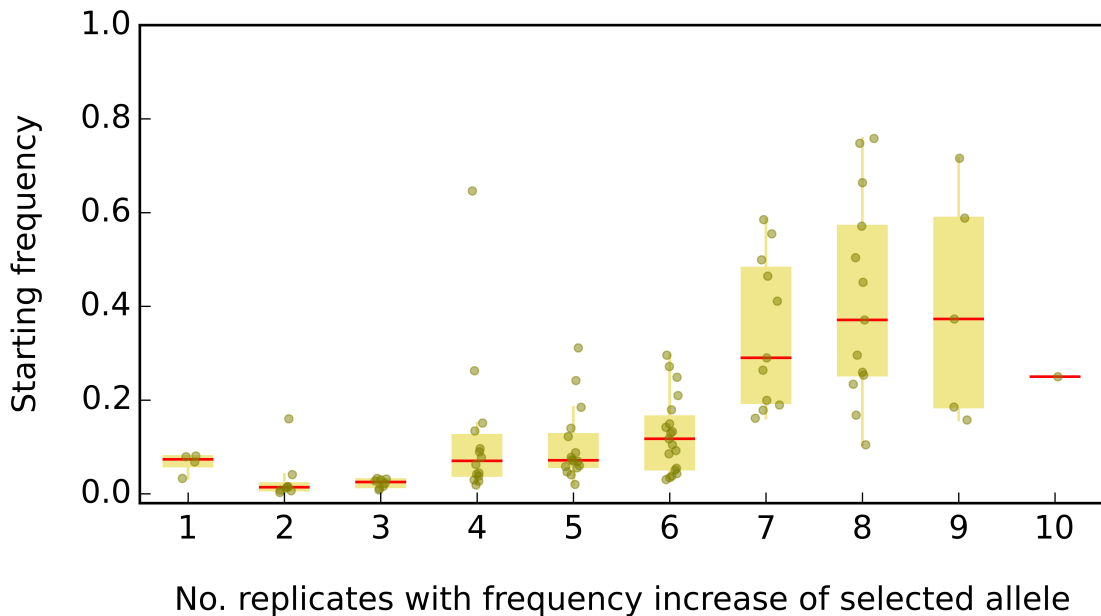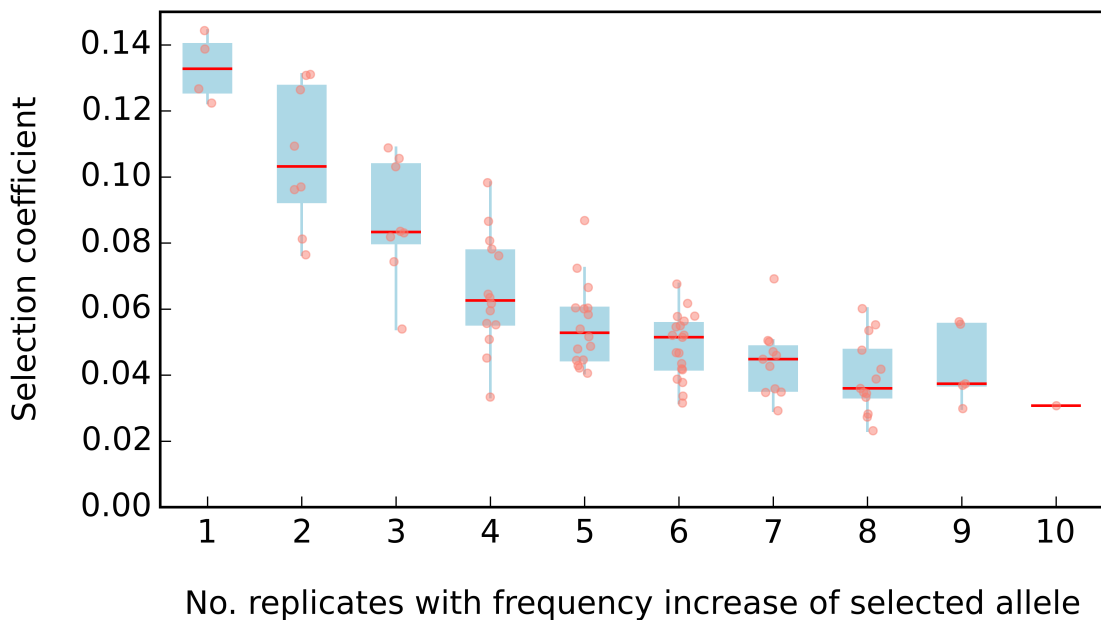

Supplement: S10 Fig — Starting frequency (top panel) and selection coefficient (bottom panel) of the selected alleles classified by the number of replicates in which a given selected allele has ≥0.1 frequency increase at generation 60 (method 1 in Materials and methods “Different approaches to determine the presence of selected alleles and their frequencies”). The selected alleles that increased in frequency (≥0.1) in only one to three replicates have low starting frequencies and the highest estimated s. Boxplots show the first and third quartile of the distribution, and horizontal bars in each box shows the median in each category. The data of individual selected alleles are shown as scattered dots in each boxplot. Data deposited in the Dryad Repository: https://doi.org/10.5061/dryad.rr137kn. (PDF) [file pbio.3000128.s010.pdf]

A

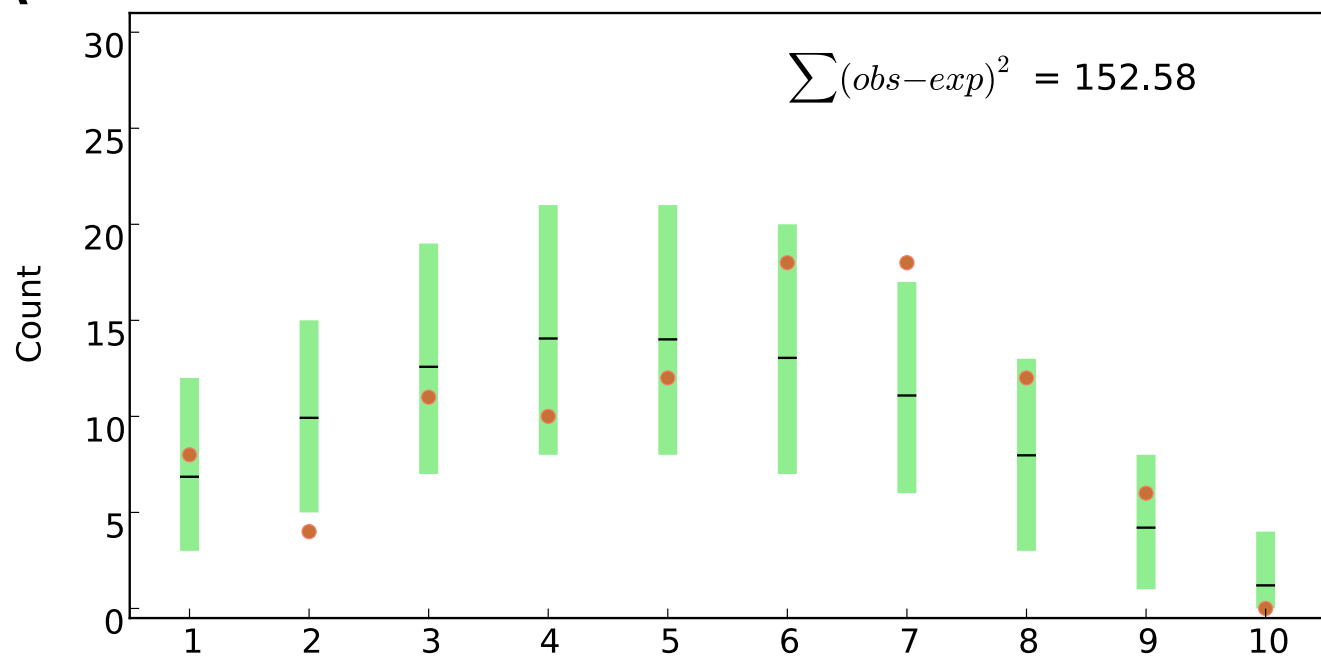

B

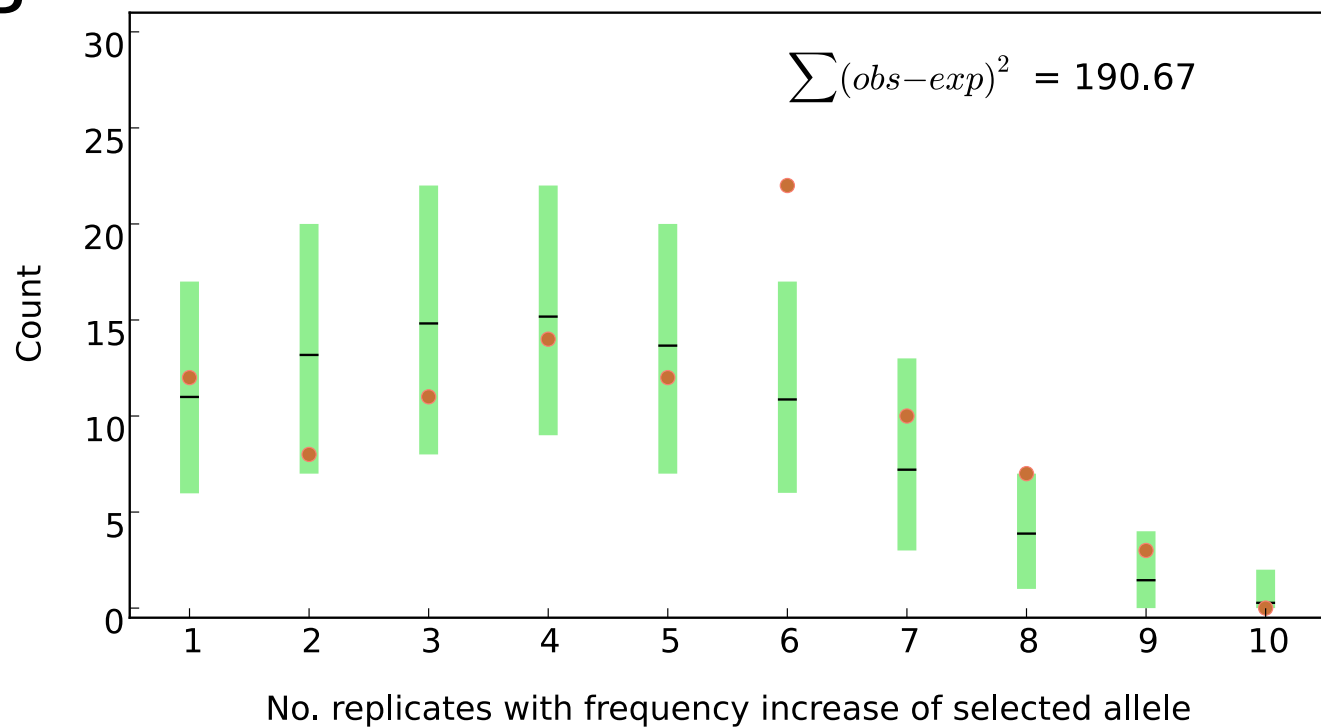

Supplement: S11 Fig — The RFS shows the frequency distribution of replicates in which selected alleles increase in frequency (threshold; A: ≥5% ASFC [method 3 in Materials and methods “Different approaches to determine the presence of selected alleles and their frequencies”]; B: ≥10% ASFC [method 4]). The RFS of experimental data (observed) is indicated by salmon dots. The expected distribution of RFS was obtained by 1,000 iterations of computer simulations (Materials and methods “D. QT paradigm without linkage”) in 10 replicates for 60 generations with 99 contributing alleles having the same starting frequency as the selected alleles in the empirical data (Fig 3A) and is indicated in light green (mean in black line). Data deposited in the Dryad Repository: https://doi.org/10.5061/dryad.rr137kn. ASFC, allele-specific frequency change; QT, quantitative trait; RFS, replicate frequency spectrum. (PDF) [file pbio.3000128.s011.pdf]

**A**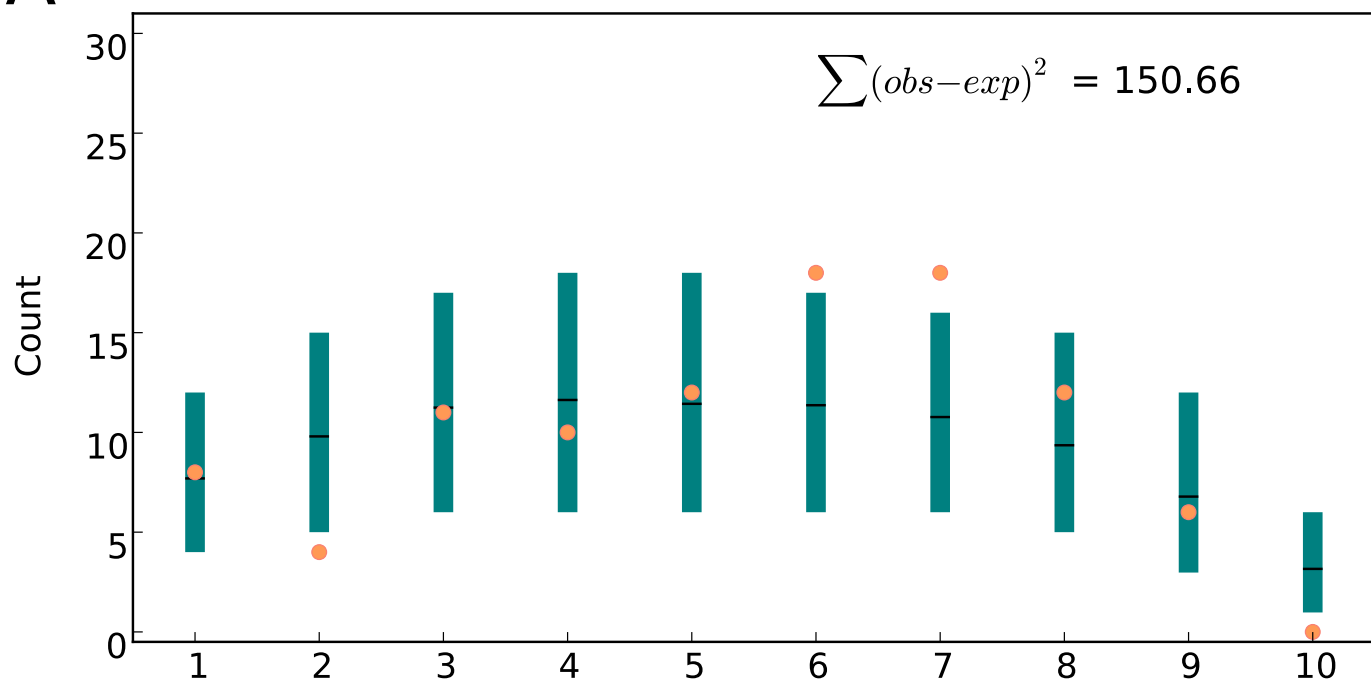**B**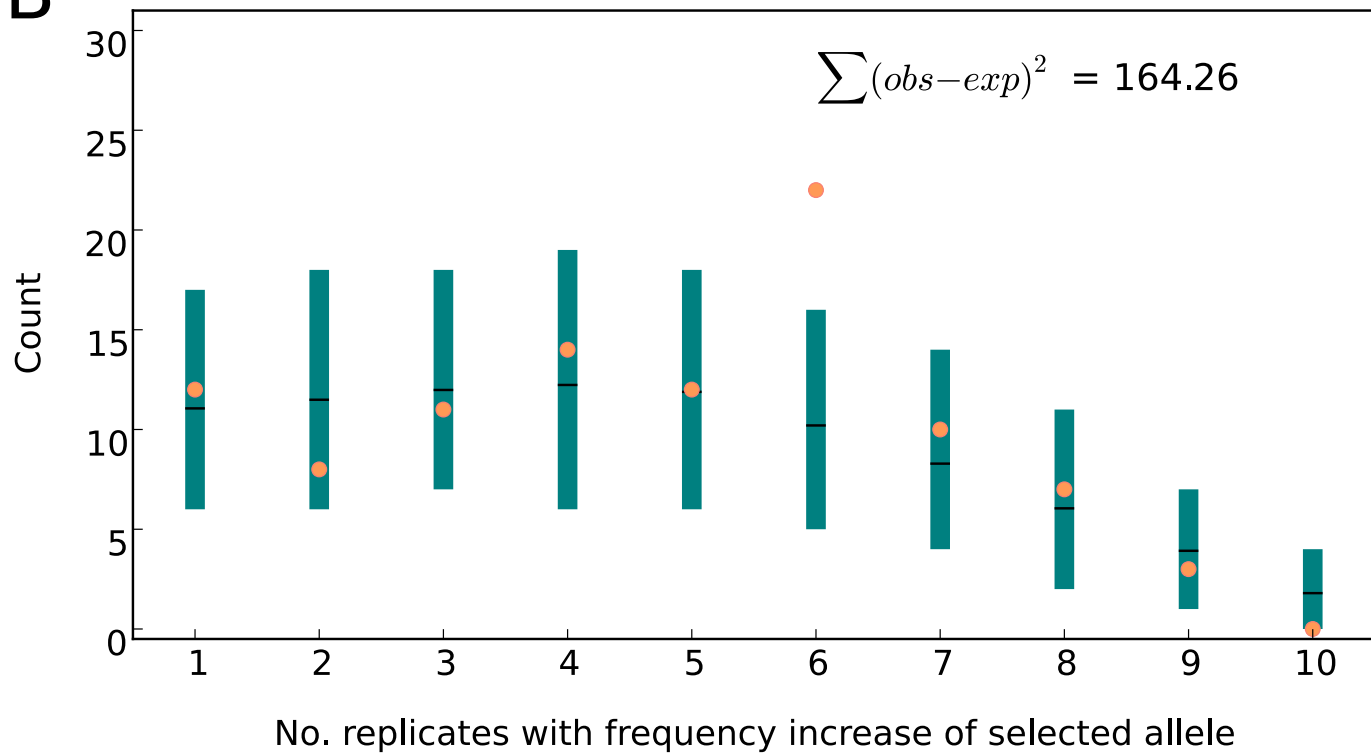

Supplement: S12 Fig — RFS shows the frequency distribution of replicates in which selected alleles increase in frequency (threshold; A: ≥5% ASFC [method 3 in Materials and methods “Different approaches to determine the presence of selected alleles and their frequencies”]; B: ≥10% ASFC [method 4]). The RFS of experimental data (observed) is indicated by salmon dots. The expected distribution of RFS was obtained by 1,000 iterations of computer simulations (Materials and methods: “E. QT paradigm with linkage”) in 10 replicates for 60 generations with 99 contributing alleles having the same starting frequency as the selected alleles in the empirical data (Fig 3A) and is indicated in dark green (mean in black line). Data deposited in the Dryad Repository: https://doi.org/10.5061/dryad.rr137kn. ASFC, allele-specific frequency change; QT, quantitative trait; RFS, replicate frequency spectrum. (PDF) [file pbio.3000128.s012.pdf]

**A**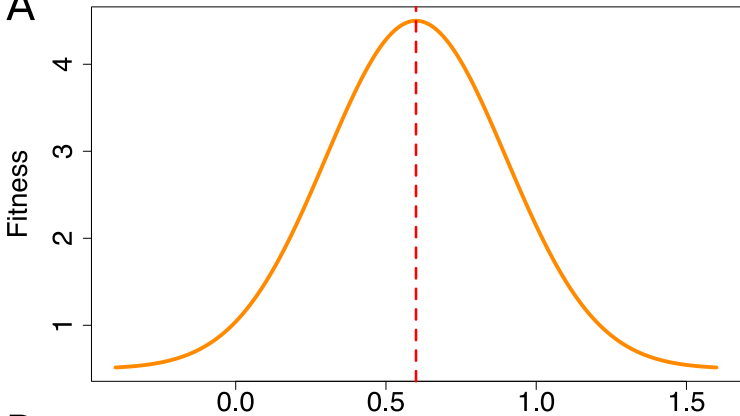**B**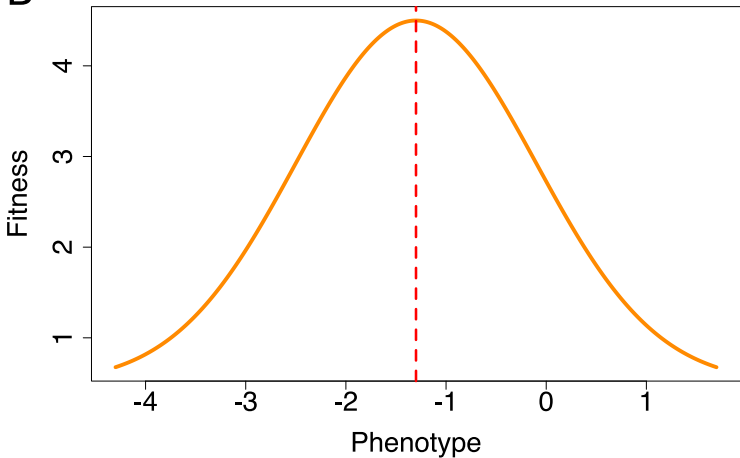

Supplement: S13 Fig — (A) Gaussian fitness function used in QT paradigm without linkage (D in S4 Fig) optimum phenotype = 0.6, standard deviation = 0.3, and fitness range from 0.5 to 4.5. (B) Gaussian fitness function used in QT paradigm with linkage (E in S4 Fig) optimum phenotype = −1.3, standard deviation = 1.2, and fitness range from 0.5 to 4.5. QT, quantitative trait. (PDF) [file pbio.3000128.s013.pdf]
