# Supplementary material for: Genetic redundancy fuels polygenic adaptation in Drosophila
Source: PLoS Biol. 2019 Feb 4;17(2):e3000128. doi: 10.1371/journal.pbio.3000128 (PMC6375663; doi:10.1371/journal.pbio.3000128)
Supplement: S4 Fig — We compare different adaptive sweep and QT scenarios to the empirical data: selective sweep simulations of alleles without (panel A) and with (panel B) linkage were studied, as well as different aspects of a QT paradigm: genetic redundancy (panel C) and simulations of AFCs assuming a QT with stabilizing selection without (panel D) and with (panel E) linkage among alleles. Sweep simulations (panel A and B) were performed for 99 (A1–6, B1–6) and 79 alleles (increasing in more than four replicates, A7 and B7). The selection coefficient (s) was estimated using the median frequency trajectories of selected alleles in replicates with ≥0.1 (method 1, orange circles) and ≥0.2 (method 2, brown circles) AFC. s was also estimated using the median frequency change in replicates with ≥5% (method 3, light blue circle) and ≥10% (method 4, dark blue circle) ASFC. s and starting frequency of the selected alleles were estimated using either all SNP characteristic of a given selected allele (“full alleles”) or only the “core region” (methods 5 and 6). See Materials and methods “Different approaches to determine the presence of selected alleles and their frequencies” for description of different methods and the definition of core region. The details of the redundancy paradigm are explained in Materials and methods “C. Genetic redundancy paradigm.” Simulations of a QT with stabilizing selection were performed with 99 loci using starting frequency of selected alleles (“full allele”) and equal effect sizes of all alleles using unlinked (panel D) or linked (panel E) alleles. AFC, allele frequency change; ASFC, allele-specific frequency change; QT, quantitative trait; SNP, single nucleotide polymorphism. (PDF) [file pbio.3000128.s004.pdf]

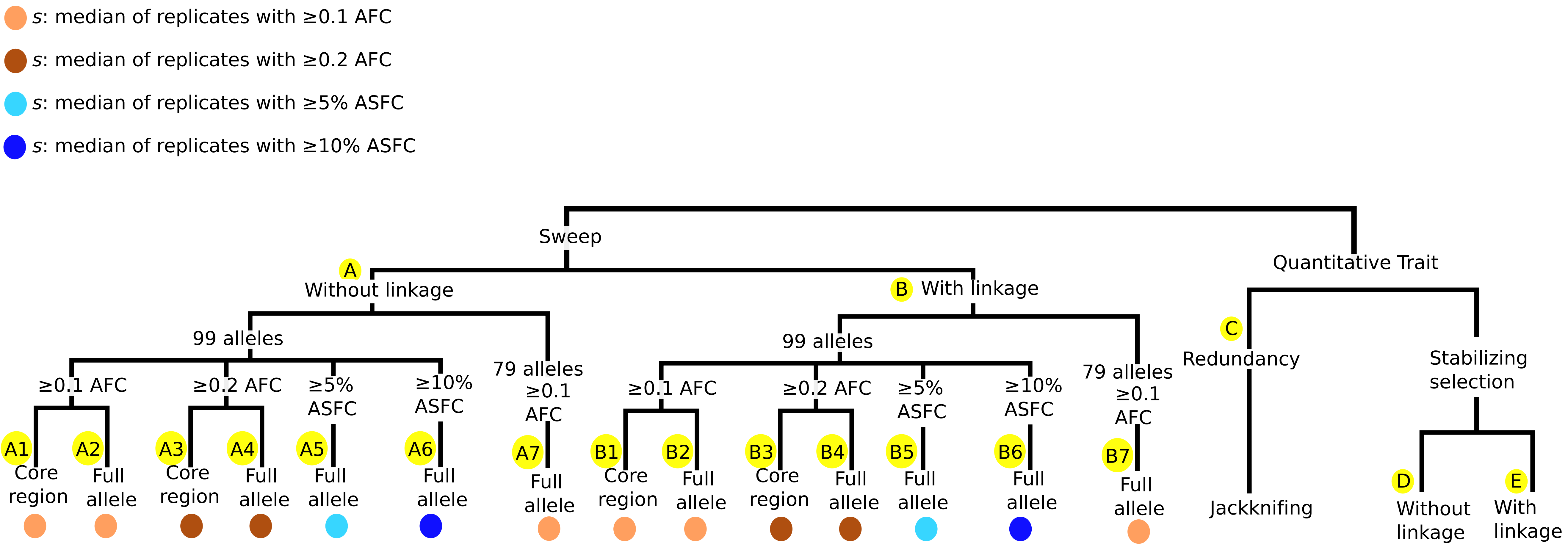
s: median of replicates with ≥0.1 AFC
s: median of replicates with ≥0.2 AFC
s: median of replicates with ≥5% ASFC
s: median of replicates with ≥10% ASFC
Sweep
Quantitative Trait
A
Without linkage
B
With linkage
99 alleles
79 alleles ≥0.1 AFC
≥0.1 AFC
≥0.2 AFC
≥5% ASFC
≥10% ASFC
A1
Core region
A2
Full allele
A3
Core region
A4
Full allele
A5
Full allele
A6
Full allele
A7
Full allele
99 alleles
79 alleles ≥0.1 AFC
≥0.1 AFC
≥0.2 AFC
≥5% ASFC
≥10% ASFC
B1
Core region
B2
Full allele
B3
Core region
B4
Full allele
B5
Full allele
B6
Full allele
B7
Full allele
C
Redundancy
Jackknifing
Stabilizing selection
D
Without linkage
E
With linkage
