# Supplementary material for: Genetic redundancy fuels polygenic adaptation in Drosophila
Source: PLoS Biol. 2019 Feb 4;17(2):e3000128. doi: 10.1371/journal.pbio.3000128 (PMC6375663; doi:10.1371/journal.pbio.3000128)
Supplement: S7 Fig — RFS shows the frequency distribution of replicates in which selected alleles increase in frequency. RFS of experimental data (observed) is indicated by salmon dots. The expected distribution of RFS was obtained by computer simulations (see Materials and methods “B. Sweep paradigm with linkage and a constant s across replicates”) and is indicated in dark gray (mean in black line). The same selection coefficients and starting frequencies were used as in S6 Fig. Data deposited in the Dryad Repository: https://doi.org/10.5061/dryad.rr137kn. RFS, replicate frequency spectrum. (PDF) [file pbio.3000128.s007.pdf]

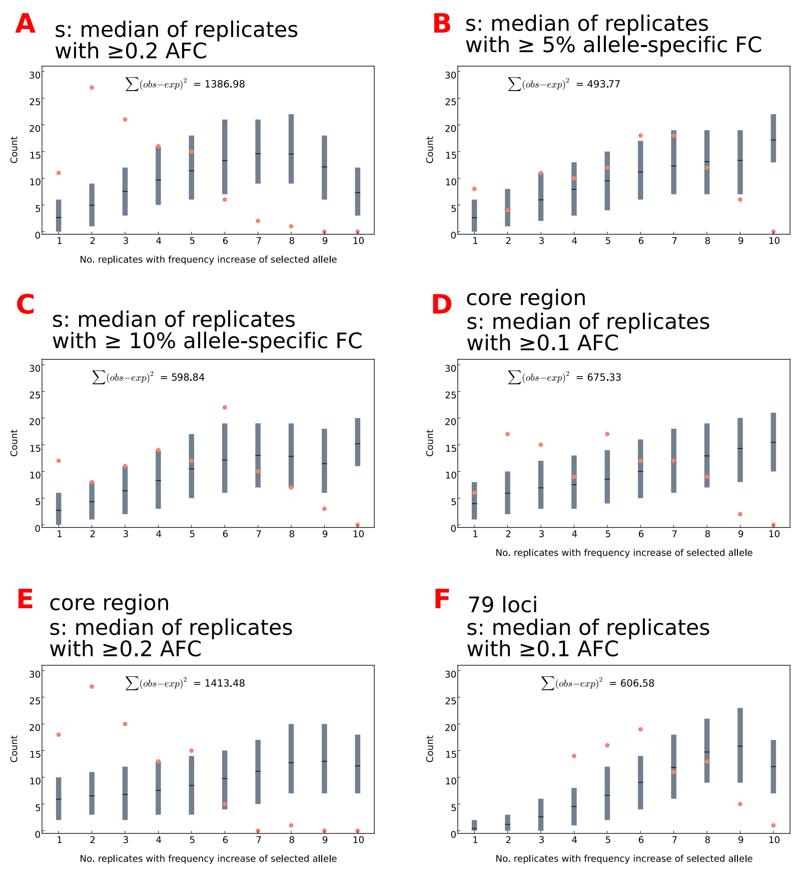

A
s: median of replicates with ≥0.2 AFC
$\sum(obs-exp)^2$ = 1386.98
B
s: median of replicates with ≥ 5% allele-specific FC
$\sum(obs-exp)^2$ = 493.77
C
s: median of replicates with ≥ 10% allele-specific FC
$\sum(obs-exp)^2$ = 598.84
D
core region s: median of replicates with ≥0.1 AFC
$\sum(obs-exp)^2$ = 675.33
E
core region s: median of replicates with ≥0.2 AFC
$\sum(obs-exp)^2$ = 1413.48
F
79 loci s: median of replicates with ≥0.1 AFC
$\sum(obs-exp)^2$ = 606.58
Count
No. replicates with frequency increase of selected allele
