# Supplementary material for: Genetic redundancy fuels polygenic adaptation in Drosophila
Source: PLoS Biol. 2019 Feb 4;17(2):e3000128. doi: 10.1371/journal.pbio.3000128 (PMC6375663; doi:10.1371/journal.pbio.3000128)
Supplement: S6 Table — (A) The size of haplotype block is used as physical or genetic distance. (B) The estimated s was computed based on the frequency trajectory of a selected allele in replicates with ≥5% and ≥10% ASFC (methods 3 and 4 in Materials and methods “Different approaches to determine the presence of selected alleles and their frequencies”). Standard errors are shown in parentheses. The table has been made using stargazer (Hlavac, Marek (2018). stargazer: Well-Formatted Regression and Summary Statistics Tables. R package version 5.2.2. https://CRAN.R-project.org/package=stargazer). ASFC, allele-specific frequency change. (DOCX) [file pbio.3000128.s019.docx]

**S6 Table Summary of regression models to identify factors affecting the estimated selection coefficient (*s*)**.

**A**

================================================================

Dependent variable:

----------------------------------

*s*

physical distance genetic distance

----------------------------------------------------------------

p0 -1.198*** -1.199***

(0.146) (0.146)

replicate frequency -0.470*** -0.471***

(0.047) (0.047)

size -0.000

(0.000)

p0^2^ 1.020*** 1.021***

(0.128) (0.128)

genetic distance -0.002

(0.002)

Constant -0.673*** -0.672***

(0.031) (0.031)

----------------------------------------------------------------

Observations 99 99

R^2^ 0.812 0.812

Adjusted R^2^ 0.804 0.804

Residual Std. Error (df = 94) 0.079 0.079

F Statistic (df = 4; 94) 101.678*** 101.556***

================================================================

Note: *p<0.1; **p<0.05; ***p<0.01

**B**

==========================================================

Dependent variable:

----------------------------

*s*

≥ 5% ASFC ≥ 10% ASFC

----------------------------------------------------------

p0 -0.126*** -0.134***

(0.047) (0.050)

replicate frequency -0.063*** -0.061***

(0.005) (0.005)

size -0.018 -0.020

(0.012) (0.012)

Constant -0.785*** -0.788***

(0.073) (0.076)

----------------------------------------------------------

Observations 99 99

R^2^ 0.666 0.617

Adjusted R^2^ 0.655 0.605

Residual Std. Error (df = 95) 0.102 0.106

F Statistic (df = 3; 95) 63.022*** 51.091***

==========================================================

Note: *p<0.1; **p<0.05; ***p<0.01

(A) The size of haplotype block is used as physical or genetic distance. (B) The estimated s was computed based on the frequency trajectory of a selected allele in replicates with ≥5% and ≥10% ASFC (methods 3 and 4 in Materials and methods “Different approaches to determine the presence of selected alleles and their frequencies”). Standard errors are shown in parentheses. The table has been made using stargazer (Hlavac, Marek (2018). stargazer: Well-Formatted Regression and Summary Statistics Tables. R package version 5.2.2. https://CRAN.R-project.org/package=stargazer). ASFC, allele-specific frequency change.
